# HCN2 Channel-Induced Rescue of Brain Teratogenesis *via* Local and Long-Range Bioelectric Repair

**DOI:** 10.3389/fncel.2020.00136

**Published:** 2020-05-26

**Authors:** Vaibhav P. Pai, Javier Cervera, Salvador Mafe, Valerie Willocq, Emma K. Lederer, Michael Levin

**Affiliations:** ^1^Allen Discovery Center at Tufts University, Medford, MA, United States; ^2^Departament de Termodinamica, Facultat de Fisica, Universitat de Valencia, Burjassot, Spain; ^3^Wyss Institute for Biologically Inspired Engineering, Harvard University, Boston, MA, United States

**Keywords:** ion channel, bioelectric, teratogen, nicotine, non-local, long-range, regenerative medicine

## Abstract

Embryonic exposure to the teratogen nicotine results in brain defects, by disrupting endogenous spatial pre patterns necessary for normal brain size and patterning. Extending prior work in *Xenopus laevis* that showed that misexpression of ion channels can rescue morphogenesis, we demonstrate and characterize a novel aspect of developmental bioelectricity: channel-dependent repair signals propagate long-range across the embryo. We show that distal HCN2 channel misexpression and distal transplants of HCN2-expressing tissue, non-cell-autonomously reverse profound defects, rescuing brain anatomy, gene expression, and learning. Moreover, such rescue can be induced by small-molecule HCN2 channel activators, even with delayed treatment initiation. We present a simple, versatile computational model of bioelectrical signaling upstream of key patterning genes such as *OTX2* and *XBF1*, which predicts long-range repair induced by ion channel activity, and experimentally validate the predictions of this model. Our results and quantitative model identify a powerful morphogenetic control mechanism that could be targeted by future regenerative medicine exploiting ion channel modulating drugs approved for human use.

## Introduction

Brain development is a paradigm case of complex organogenesis, requiring precise control of neural progenitor cell behaviors (Stanger, 2008; Joseph and Hermanson, 2010). The dorsal ectoderm differentiates into the neural plate, with the anterior region forming the brain under the influence of positive and negative effectors (De Robertis and Kuroda, 2004; Stern, 2005). Physical forces (Thompson, 1942; Stanger, 2008), gene regulatory networks (Zhao et al., 2011), and bioelectric signals (Pai et al., 2015a,b; Smith et al., 2018) regulate this complex process.

The membrane voltage potential is a fundamental property of all cells. Endogenous ion fluxes and membrane voltage patterns in embryonic and somatic cells regulate cell behavior and organ-level patterning across eukaryotes (Nuccitelli, 2003; Bates, 2015; Humphries et al., 2017; Levin and Martyniuk, 2018). They also play important roles in directing large scale growth and form *in vivo*, including tissue/organ regeneration (Tseng and Levin, 2012; Beane et al., 2013; Perathoner et al., 2014), left-right patterning (Levin et al., 2002; Aw et al., 2010; Pai et al., 2017), craniofacial morphogenesis (Adams et al., 2016; Belus et al., 2018), heart and muscle patterning (Lobikin et al., 2015; Pitcairn et al., 2017), and CNS patterning (Ribera, 1999; Lange et al., 2011; Aprea and Calegari, 2012; Pai et al., 2012a,b, 2015b; Avila et al., 2013; Sequerra et al., 2018; Smith et al., 2018).

Endogenous spatiotemporal patterns of cellular resting potentials regulate neural tissue induction and neural progenitor apoptosis and proliferation in the developing brain, as revealed by loss-of-function experiments in model systems (Pai et al., 2015a,b) and by naturally-occurring genetic channelopathies (Smith et al., 2018). Moreover, establishing correct spatial membrane voltage pre patterns *via* misexpression of specific channels to control membrane voltage can rescue neural patterning defects caused by aberrant Notch signaling, mechanical damage, or exposure to neuroteratogens, and normalize the expression of canonical brain patterning genes (Pai et al., 2015b, 2018; Herrera-Rincon et al., 2017). Changes in membrane voltage are transduced *via* gap-junctional signaling and calcium dynamics to control the expression of crucial transcription factors necessary for brain patterning (Pai et al., 2015b).

Hyperpolarization-activated Cyclic nucleotide-gated (HCN) channels are voltage-gated channels, with a threshold voltage that is affected by metabolic state (Biel et al., 2009; Wahl-Schott and Biel, 2009; Benarroch, 2013). The HCN channel family includes variants HCN1-4, all of which are known to be expressed in embryonic cells and early *Xenopus* embryos (Yasui et al., 2001; Qu et al., 2008; Vicente-Steijn et al., 2011; Spater et al., 2013; Session et al., 2016; Pai et al., 2017, 2018; Pitcairn et al., 2017). However, their roles in embryonic development and possible utility as therapeutic targets are largely unexplored (Postea and Biel, 2011; Benarroch, 2013).

Misexpression of HCN2 channels in *Xenopus* embryos restored the bioelectric prepattern and brain patterning disrupted by nicotine (Pai et al., 2018). We characterized in detail how nicotine’s teratogenic effects occur *via* the disruption of endogenous bioelectric patterns and presented a quantitative model of the physiology of the affected cells and their rescue under HCN2 dynamics (Pai et al., 2018). Here, we use this established neuroteratogen (Slotkin et al., 2005; Huizink and Mulder, 2006; Slotkin, 2008; Velazquez-Ulloa, 2017) to ask important new questions about the spatial properties of non-cell-autonomous bioelectric controls of brain patterning and test the hypothesis that repair can be induced without the need for gene therapy. Remarkably, we found that HCN2-activated repair can be triggered at considerable distance from the brain. We constructed and tested a computational model which explains how repair-inducing bioelectric states can propagate across tissues. Lastly, we found that FDA-approved small-molecule drugs targeting ion channels can induce brain repair, counteracting teratogenic exposure without the need for transgenes. Together, the predictive computational model of bioelectric signal propagation across tissues and the functional data identify a novel long-range regulator of brain organogenesis and suggest molecular bioelectric strategies as interventions to induce repair in a roadmap for regenerative applications targeting birth defects (Mathews and Levin, 2018; McLaughlin and Levin, 2018).

## Materials and Methods

### Animal Husbandry

*Xenopus laevis* embryos were fertilized *in vitro* according to standard protocols in 0.1× Marc’s Modified Ringer’s (MMR; 10 mM Na^+^, 0.2 mM K^+^, 10.5 mM Cl^−^, 0.2 mM Ca^2+^, pH 7.8; Sive et al., 2000). *Xenopus* embryos of both sexes were housed at 14–18°C and staged according to Nieuwkoop and Faber (1958). For animals used in behavior trials, individuals of both sexes were raised under 12 h:12 h light:dark cycle at a temperature of 16°C at no more than 30 individuals per 100 × 25 mm Petri dish. After stage 46, tadpoles were fed twice per day on standard sera micron powdered food until behavioral testing. All experiments were approved by the Tufts University Animal Research Committee (M2017-53) following the guide for the care and use of laboratory animals.

### Microinjections

Capped synthetic mRNAs generated using the mMessage mMachine kit (Ambion) were dissolved in nuclease-free water and injected into embryos immersed in 3% Ficoll using standard methods (Sive et al., 2000). Each injection delivered between 0.5–1 ng of mRNA (per blastomere) into the embryos at the indicated stages into the middle of a cell in the animal hemisphere. *Hcn2-WT* and *Hcn2-DN* were mammalian (mouse) hyperpolarization-activated cyclic nucleotide-gated channel 2, modified as detailed in Pai et al. (2018).

### Drug Exposure

Embryos were incubated in chemicals dissolved in 0.1× MMR during the stages of interest as indicated in the respective experiments followed by several washes with 0.1× MMR. Embryos were exposed to 0.1 mg/ml nicotine (sigma—N3876) from stage 10–35 unless otherwise specified (targeting neurodevelopment while allowing normal cleavage and gastrulation). Embryos were exposed to 200 μM lamotrigine (tocris—2289) and 175 μM gabapentin (tocris—0806) at the specified stages. The dose of lamotrigine and gabapentin drugs were titrated to a level at which no general toxicity was observed, and the survival rate of embryos was similar to untreated controls (Supplementary Figure S1).

### Morphometrics

Tadpoles used for morphometric analysis were imaged with a Nikon SMZ1500 microscope with a Retiga 2000R camera and Q-capture imaging software. Landmarks were chosen and annotated using ImageJ software (Schneider et al., 2012) based on biological relevance and reproducibility across tadpoles with the varying brain and head morphologies: (1) anterior tip of the head; (2) space between the olfactory bulbs at the beginning of the forebrain; (3) center of the transition line between forebrain and midbrain; (4) center of the transition line between midbrain and hindbrain; (5) transition point between the hindbrain and spinal cord, and (6); and (7) lateral-most points of the head at the eyes. MorphoJ (Klingenberg, 2011) was used for Canonical Variate Analysis to quantify and graphically represent changes in brain regions relative to head shape. MorphoJ was also used to calculate Procrustes distances and perform statistical analysis. Our analysis was conservative, as nicotine-treated embryos that had such severe defects that we could not identify the landmarks were excluded.

### Associative Learning Behavior Test at Stage 48

Tadpoles were fed directly before trials and provided food ad-lib during the trials. All behavioral trials involved a color-based associative learning assay, performed as previously described, using an automated behavior analysis platform (Blackiston et al., 2010a; Blackiston and Levin, 2012). Briefly, individual tadpoles were introduced to the chamber which was half-illuminated with red light and half blue light to probe their innate color preferences for 30 min in the absence of any punishment. In the learning acquisition phase, each tadpole received a 1.2 mA shock whenever it occupied the red area. This duration was 20 min, with colors in the chamber being inverted every 5 min to ensure that immobility is not scored as a success. Then, tadpoles were given a 90 min rest in which the entire chamber is illuminated with blue light and no shock is delivered. Learning was scored by giving the tadpoles a choice between red and blue light for 5 min with no punishment. The entire block of acquisition-rest-probe was repeated six times. A tadpole was determined to have learned if their preference for a red light was below 40% as averaged across the final three probe sessions of the experiment.

### *In situ* Hybridization

*Xenopus* embryos were collected and fixed in MEMFA (1 h at room temperature; Sive et al., 2000), and *in situ* hybridization was performed as previously described (Sive et al., 2000). *in situ* antisense probes were generated *in vitro* from linearized templates using a DIG (Digoxigenin)-labeling mix (Roche). Chromogenic reaction times were optimized for signal-to-background ratio. Probes used were *otx2* (Pannese et al., 1995) and *xbf1* (Eagleson and Theisen, 2008). A stock solution of each probe was used for all experimental groups, which were incubated together for the same amount of reaction time, to avoid any variability due to probe concentration or length of reaction time.

### Imaging V_mem_ Using CC2-DMPE:DiBAC_4_(3)

CC2-DMPE and DiBAC4(3) ratiometric voltage reporter dyes were obtained from Invitrogen and used as per the standard protocol (Adams and Levin, 2013). Briefly, CC2-DMPE stock (5 mM) was dissolved 1:1,000 in 0.1× MMR and the embryos were incubated in the dark in this solution for at least 1 h followed by five washes with 0.1× MMR. DiBAC_4_(3) stock (1.9 mM) was dissolved 1:1,000 in 0.1× MMR and the CC2-DMPE-stained embryos were then incubated in the dark in this solution for at least 30 min washed thoroughly in 0.1× MMR followed by visualization under the microscope. An Olympus BX-61 microscope equipped with a Hamamatsu ORCA AG CCD camera and controlled by MetaMorph software (Molecular Devices), was used to collect images. ImageJ was used to quantify the fluorescence intensities of the CC2-DMPE: DiBAC signal along the red line across the image as indicated in the illustrations in Figures 4G, 9E). Fluorescence values at each point along this line were used to plot graphs.

**Figure 1:**
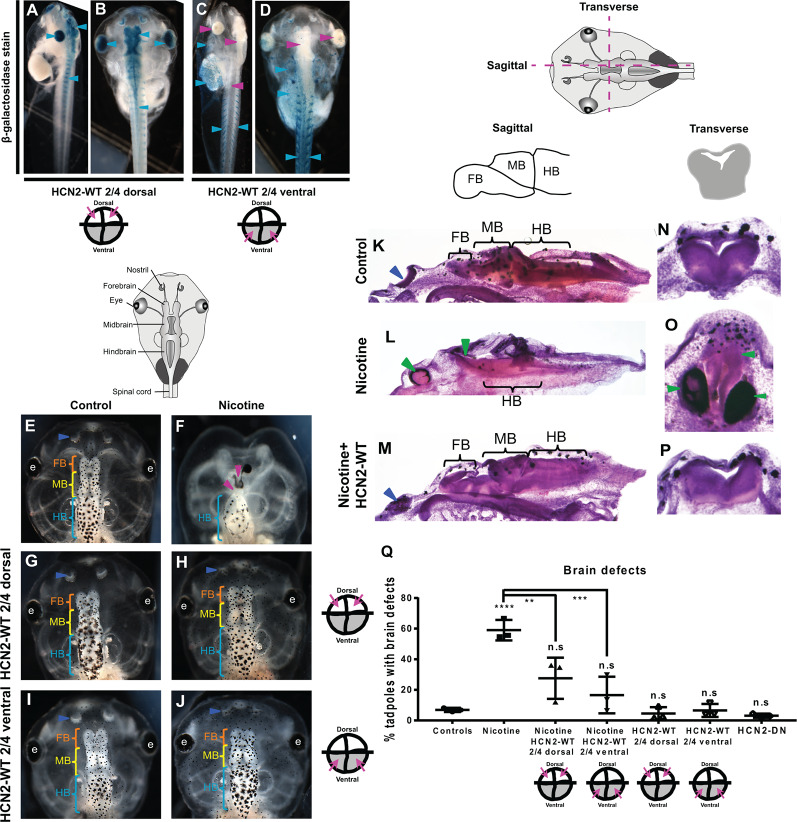
Both local (CNS) and distant (non-CNS) expression of HCN2 channels rescues nicotine-induced brain morphology defects in *Xenopus* embryos. **(A–J)** Representative images of stage 45 tadpoles. **(A–D)** β-galactosidase expression assessed using X-Gal (blue) in bleached tadpole co-injected with *Hcn2-WT* and *β-galactosidase* mRNA. β-galactosidase was observed mainly in the eye, brain, and spinal cord (cyan arrowheads), of dorsal blastomere injections. In ventral blastomere injections, β-galactosidase was absent from the eye, brain, and spinal cord (magenta arrowheads) and was mainly present in the brachial arches, gut, heart, and muscles (cyan arrowheads). **(E–J)** Control (untreated and uninjected) or nicotine-treated tadpoles with or without microinjection with *Hcn2-WT* mRNA either in dorsal or ventral blastomeres at the four-cell stage. Blue arrowheads indicate intact nostrils, orange brackets indicate intact forebrain (FB), yellow brackets indicate intact midbrain (MB), cyan brackets indicate intact hindbrain (HB), intact eyes (e), and magenta arrowheads indicate severe brain morphology defects. **(K–P)** Histological staining (hematoxylin and eosin) of agarose sections through the brain (sagittal and transverse sections through midbrain) of stage 45 tadpoles. Control (untreated and uninjected) or nicotine-treated tadpoles with or without microinjection with *Hcn2-WT* mRNA in the ventral blastomeres at the four-cell stage. FB-forebrain, MB-midbrain, HB-hindbrain, blue arrowheads indicate intact nostrils, and green arrowheads indicate severe brain morphology defects. **(Q)** Quantification of stage 45 tadpole brain morphology defects under indicated conditions. Percentage of tadpoles with brain defects for each experimental group are Controls—7%, Nicotine—59%, Nicotine+HCN2-WT 2/4 dorsal—27%, Nicotine+HCN2 2/4 ventral—16%, HCN2 2/4 dorsal—5%, HCN2 2/4 ventral—6%, and HCN2-DN—3%. Data are mean ± SD, ****p < 0.0001, ***p < 0.001, **p < 0.01, n.s.: non-significant (one-way ANOVA with Tukey’s *post hoc* test for *n* = 3 independent experiments with *N* > 50 embryos per treatment group per experiment).

**Figure 2:**
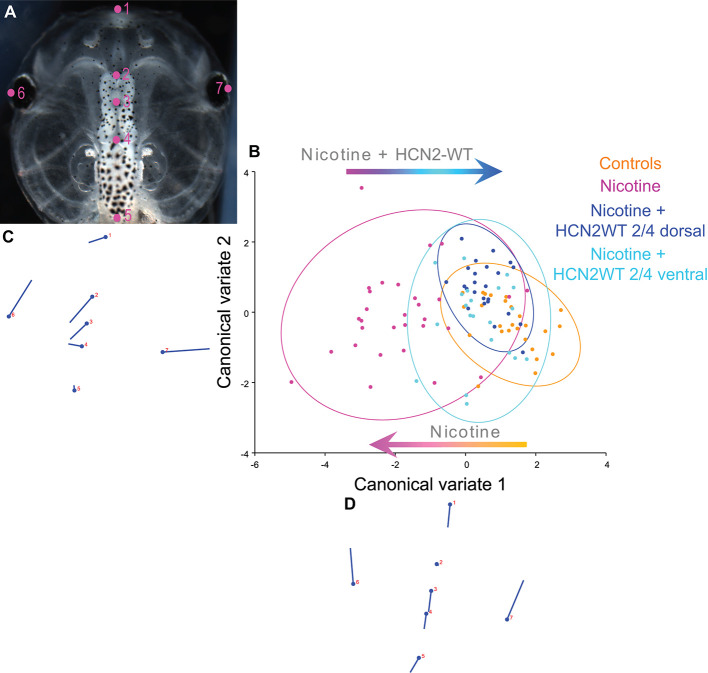
Both local (CNS) and distant (non-CNS) expression of HCN2 channels restore brain size in proportion to head shape in nicotine-exposed embryos. **(A–D)** Morphometrics canonical variate analysis of brain size in proportion to head shape of stage 45 tadpoles. Representative image of a stage 45 control tadpole **(A)** illustrating the seven landmarks used in this analysis. **(B)** Canonical variate analysis, showing confidence ellipses for means at a 0.95 probability of shape data. Confidence ellipses are colored to correspond with treatment as indicated. *N* > 20 for each group. Procrustes distances: Control vs. nicotine = 0.1, Control vs. nicotine+dorsal-HCN2-WT = 0.04, Control vs. nicotine+ventral-HCN2-WT = 0.05. ANOVA of centroid shape between the controls, nicotine treatment, and nicotine+HCN2-WT (both dorsal and ventral) showed significant differences between the groupings (dorsal microinjection—*F* = 10.67, *p* < 0.0001; ventral microinjection—*F* = 9.0, *p* < 0.0001). **(C,D)** Canonical variate axis legend showing the movement of each of the seven landmarks. Each ball represents the landmark as indicated by the number and the accompanying stick represents the direction and extent of movement of that particular landmark.

**Figure 3:**
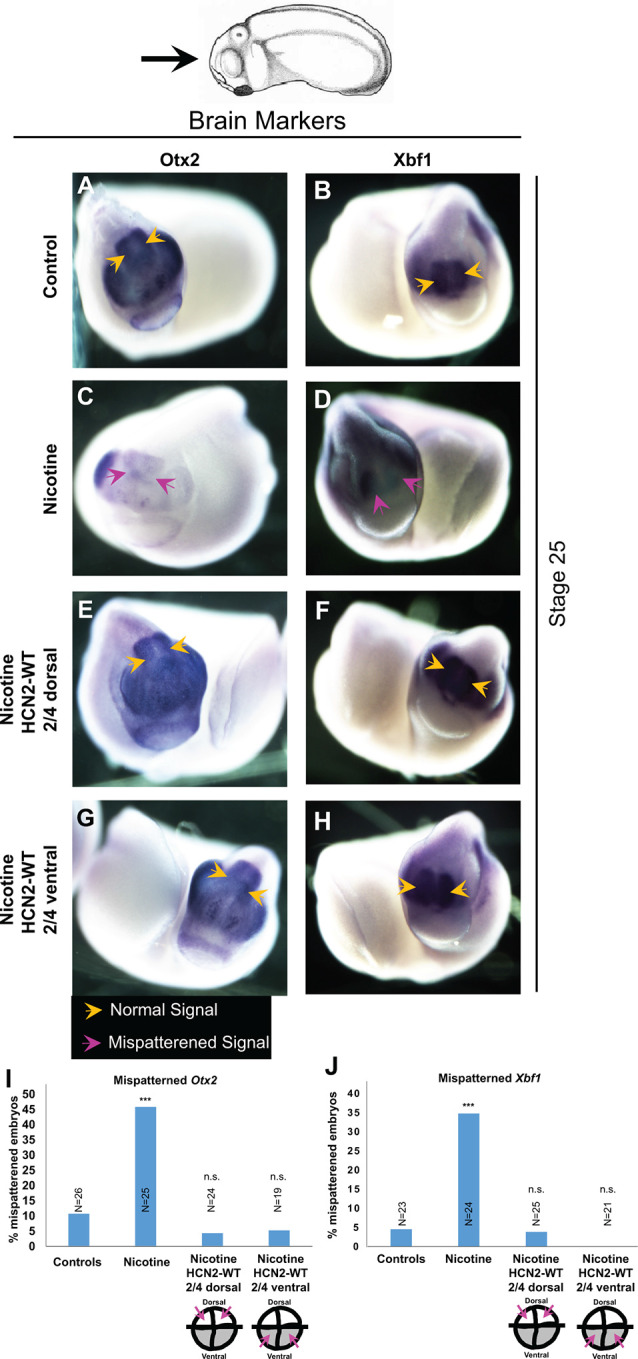
Both local (CNS) and distant (non-CNS) HCN2 channel expression restore nicotine exposure-induced mispatterning of brain markers during neural development. **(A–H)** Representative images of stage 25 embryos as illustrated with the angle of view marked by the black arrow. *in situ* hybridization with digoxigenin-labeled antisense, riboprobes were used to detect *Otx2* and *Xbf1* in the indicated experimental groups. Yellow arrows indicate normal expression pattern and magenta arrows indicate significantly mispatterned expression. **(I,J)** Quantification of *in situ* hybridized embryos for *Otx2* (*N* ≥ 25 embryos for each experimental group) and *Xbf1* (*N* ≥ 20 embryos for each experimental group) from *n* = 3 independent experiments pooled together. Data are represented as mean with *χ*^2^ squared test for differences in proportions, ***p < 0.001, n.s.: non-significant.

**Figure 4:**
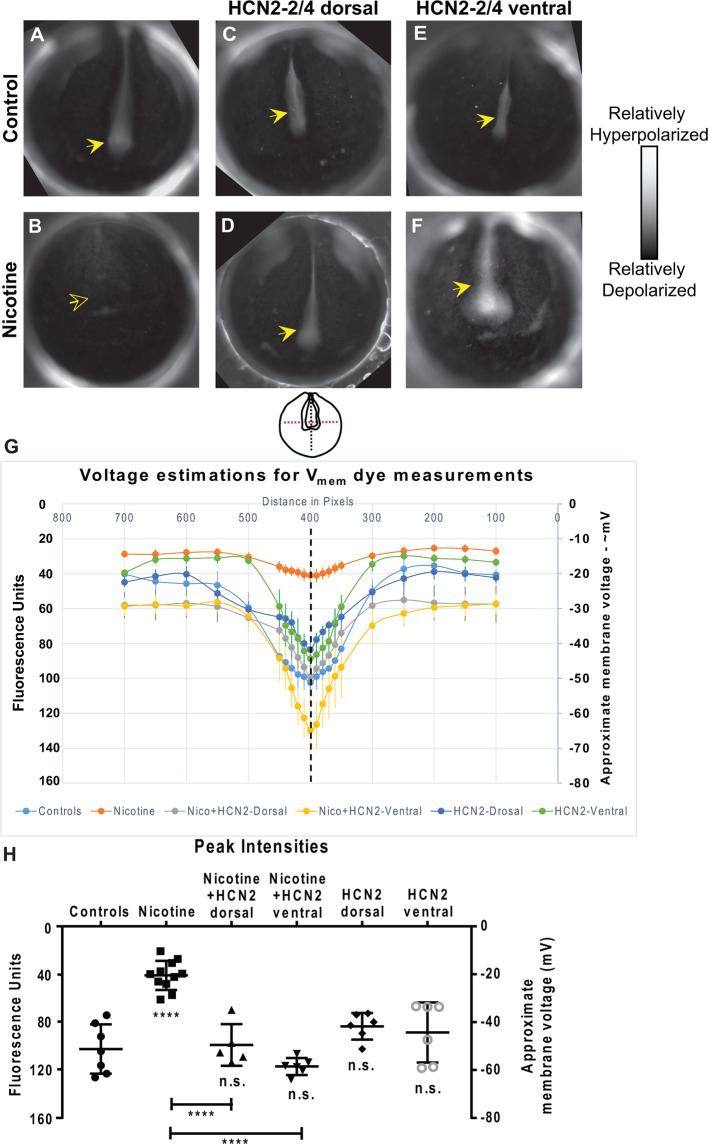
Both local (CNS) and distant (non-CNS) expression of HCN2 channels restore neural developmental membrane voltage prepatterns in nicotine-exposed embryos. **(A–F)** Representative CC2-DMPE membrane voltage reporter dye images of stage ~15 *Xenopus* embryos: Control (untreated and uninjected) or nicotine-treated embryos with or without microinjection with *Hcn2-WT* mRNA either in dorsal or ventral blastomeres at the four-cell stage. Solid yellow arrows indicate characteristic hyperpolarization in the neural plate as previously reported (Pai et al., 2015b, 2018). Hollow yellow arrows indicate significantly reduced signal (depolarization) within the neural plate in comparison to controls. **(G,H)** Quantification of fluorescence from CC2-DMPE images of stage 15 *Xenopus* embryos along with electrophysiology-based membrane voltage approximations [as previously reported in refs (Pai et al., 2015b, 2018)] for the indicated conditions. **(G)** Quantification obtained along the magenta dotted line indicated in the illustration. **(H)** Quantification at the point of intersection of the magenta and black dotted line indicated in the illustration. Data represented as mean ± SD, ****p < 0.0001. n.s.: non-significant (one-way ANOVA with Tukey’s *post hoc* test for *N* > 5 embryos for each treatment group at each point of the indicated spatial distance).

**Figure 5:**
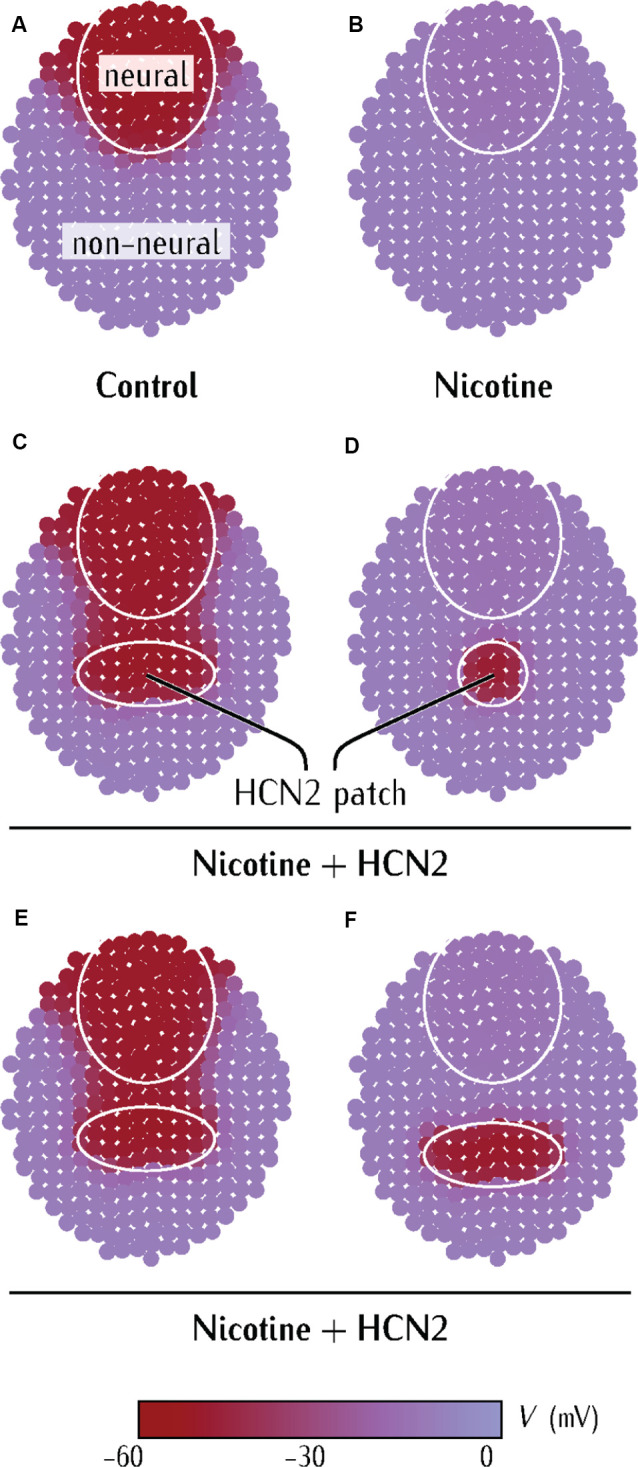
Model predicts limiting conditions for distant (non-CNS) HCN2 channel-mediated rescue of membrane voltage prepattern in nicotine-exposed embryos. **(A–F)** Simulations from a physiological model of neurulating *Xenopus* embryo as detailed in Supplementary Figures S1–S3. Maroon color represents the region of polarized membrane voltage. Purple color represents the region of depolarized membrane voltage. The pattern in **(A)** is analogous to the membrane voltage pattern seen in *Xenopus* embryos with voltage reporter dyes (Pai et al., 2015b).

**Figure 6:**
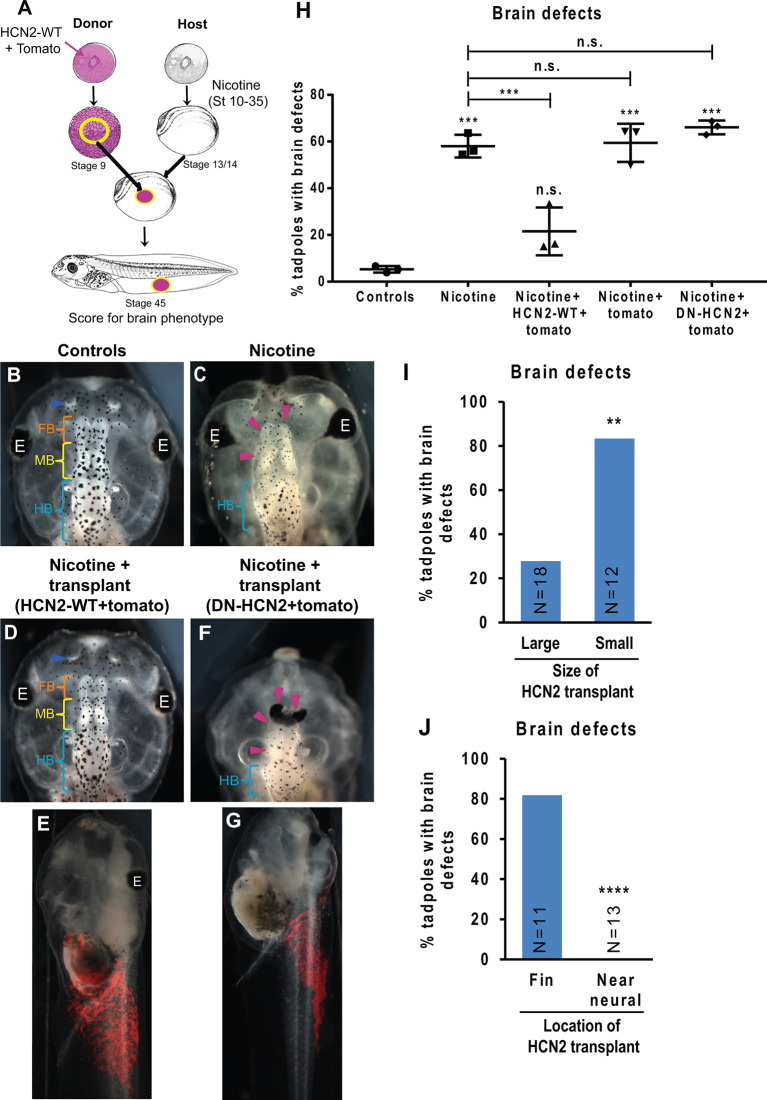
HCN2 donor tissue can rescue brain patterning in nicotine-exposed recipient embryos over long distances. **(A)** *Xenopus* animal cap transplant experimental setup. The excised animal caps were transplanted into age-matched sibling embryos. **(B–G)** Representative images of stage 45 tadpoles from transplant receiving host for indicated treatment conditions. Blue arrowheads indicate intact nostrils, orange brackets indicate intact forebrain (FB), yellow brackets indicate intact midbrain (MB), cyan brackets indicate intact hindbrain (HB), and magenta arrowheads indicate severe brain morphology defects. The red region in **(E)** and **(G)** is the tomato tracer indicating the transplanted graft. **(H)** Quantification of stage 45 tadpole brain morphology defects under indicated conditions. Percentage of tadpoles with brain defects for each experimental group are Controls—5%, Nicotine—58%, Nicotine+HCN2-WT-tomato—22%, Nicotine+tomato—59%, and Nicotine+DN-HCN2-tomato—65%. Data are mean ± SD, ***p < 0.001, n.s.: non-significant (one-way ANOVA with Tukey’s *post hoc* test for *n* = 3 experiments with *N* > 20 host embryos per treatment group per experiment). **(I,J)** Quantification of brain morphology defects in stage 45 tadpoles exposed to nicotine and receiving *Hcn2-WT+tomato* mRNA expressing tissue transplant. The tadpoles were either sorted by the size of the transplant **(I)** or location of transplant **(J)** based on the tomato signal (red) at stage 45. Percentage of tadpoles with brain defects for each experimental group are: Large—27%, Small—83%, Fin—81%, and Near neural—0%. Data pooled from *n* = 3 independent experiments are represented as mean with *χ*^2^ squared test for differences in proportions, ****p < 0.0001, **p < 0.01.

**Figure 7:**
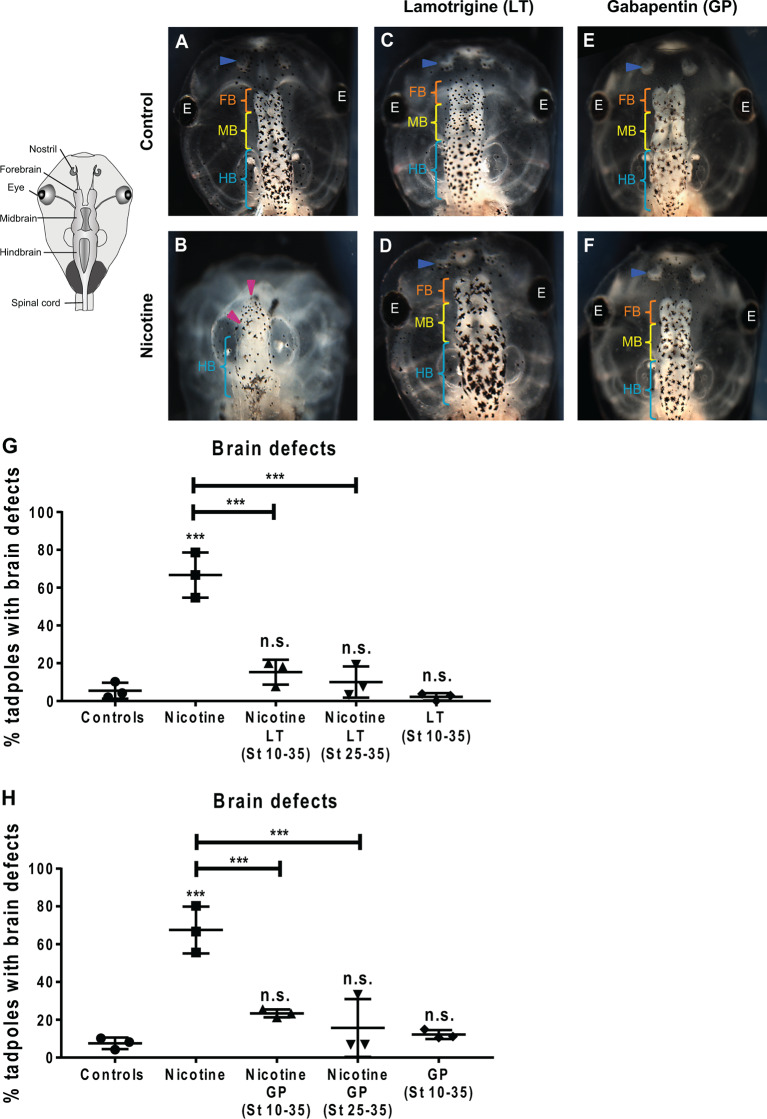
Non-local exposure to Lamotrigine and Gabapentin (HCN channel agonist) rescues nicotine-induced brain morphology defects in *Xenopus* embryos. **(A—F)** Representative images of stage 45 tadpoles. Control (untreated and uninjected) or nicotine-treated tadpoles with or without treatment with lamotrigine (LT, stage 10–35) or gabapentin (GP, stage 10–35). Blue arrowheads indicate intact nostrils, orange brackets indicate intact forebrain (FB), yellow brackets indicate intact midbrain (MB), cyan brackets indicate intact hindbrain (HB), and magenta arrowheads indicate severe brain morphology defects. **(G,H)** Quantification of stage 45 tadpole brain morphology defects under the indicated conditions for lamotrigine (LT; **G**) or gabapentin (GP; **H**). Percentage of tadpoles with brain defects for each experimental group are: **(G)** Controls—5%, Nicotine—67%, Nicotine+LT (St10–35)—15%, Nicotine+LT (St25–35)—10%, and LT(St 10–35)—2%; (**H**) Controls—7%, Nicotine—68%, Nicotine+GP (St10–35)—23%, Nicotine+GP(St25–35)—16%, and GP(St 10–35)—11%. Data are mean ± SD, ***p < 0.001, n.s.: non-significant (one-way ANOVA with Tukey’s *post hoc* test for *n* = 3 independent experiments with *N* > 50 embryos per treatment group per experiment).

**Figure 8:**
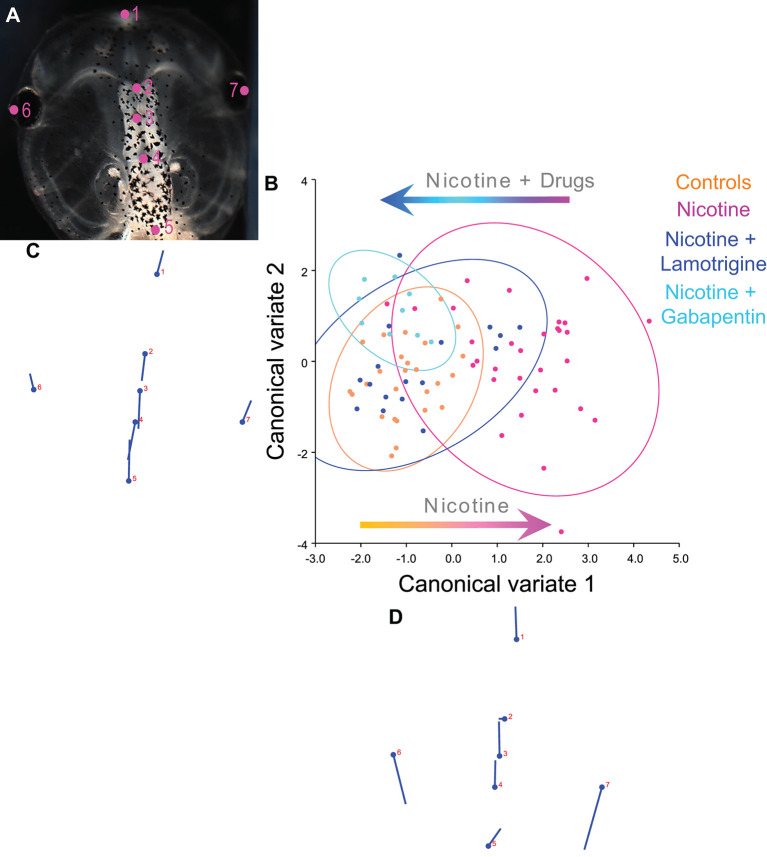
Lamotrigine and Gabapentin restore the brain size relation to anterior head shape in nicotine-exposed embryos. **(A–D)** Morphometrics canonical variate analysis of brain size in proportion to head shape of stage 45 tadpoles. Representative image of a stage 45 control tadpole **(A)** illustrating the seven landmarks used in this analysis. **(B)** Canonical variate analysis, showing confidence ellipses for means at a 0.95 probability of shape data. Confidence ellipses are colored to correspond with treatment as indicated. Lamotrigine and gabapentin treatments were from stage 10–35. *N* > 10 for each group. Procrustes distances: Control vs. nicotine = 0.12, control vs. nicotine+lamotrigine = 0.04, control vs. nicotine+gabapentin = 0.06. ANOVA of centroid shape between the controls, nicotine+lamotrigine, and nicotine+gabapentin in relation to nicotine treatment confirmed significant differences between the groups (*F* = 8.2, *p* < 0.0001). **(C,D)** Canonical variate axis legend showing the movement of each of the seven landmarks. Each ball represents the landmark as indicated by the number and the accompanying stick represents the direction and extent of movement of that particular landmark.

**Figure 9:**
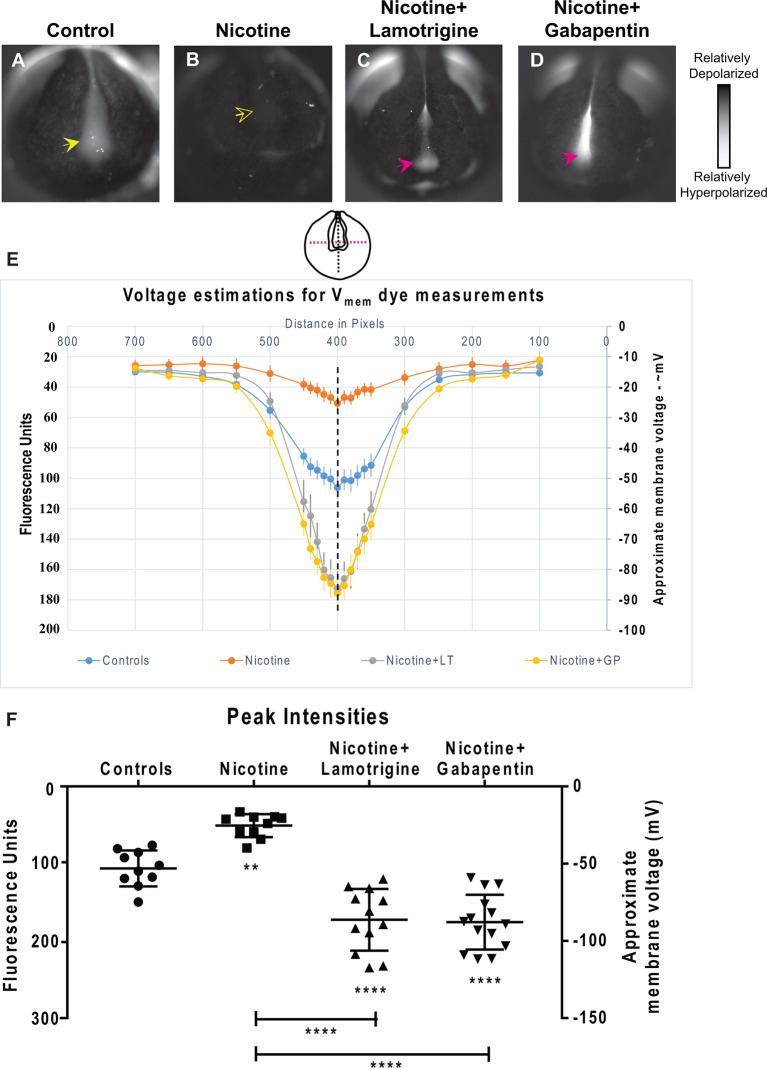
Lamotrigine and Gabapentin restore neural development membrane voltage prepattern in nicotine-exposed embryos. **(A–D)** Representative CC2-DMPE membrane voltage reporter dye images of stage ~15 *Xenopus* embryos: Control (untreated and uninjected) and nicotine-treated embryos with or without lamotrigine (stage 10 onwards) or gabapentin (stage 10 onwards) treatment. Solid yellow arrows indicate characteristic hyperpolarization in the neural plate as previously reported (Pai et al., 2015b, 2018). Hollow yellow arrows indicate significantly reduced signal (depolarization) within the neural plate in comparison to controls. Magenta arrows indicate significantly enhanced hyperpolarization in the neural plate compared to controls. **(E,F)** Quantification of fluorescence from CC2-DMPE images of stage ~15 *Xenopus* embryos along with electrophysiology-based membrane voltage approximations [as previously reported in references (Pai et al., 2015b, 2018)] for the indicated conditions (LT-lamotrigine, GP-gabapentin). **(E)** Quantification obtained along the magenta dotted line indicated in the illustration. **(F)** Quantification at the point of intersection of the magenta and black dotted line indicated in the illustration. Data represented as mean ± SD, **p < 0.01, ****p < 0.0001 (one-way ANOVA with Tukey’s *post hoc* test for *N* > 10 embryos for each treatment group at each point of the indicated spatial distance).

### Microsurgery

Transplants were performed as previously described (Viczian and Zuber, 2010). Animal caps were excised from tdTomato-expressing stage 9 donors in 0.75× MMR and were allowed to heal overnight in 0.75× MMR at 14°C. Sibling uninjected host embryos were treated with nicotine (0.1 ml/ml) beginning stage 10 and incubated overnight at 14°C. At stage 13, the vitelline membranes of nicotine-exposed recipient embryos were removed in 0.1× MMR+nicotine (0.1 mg/ml). A small square piece of tissue was removed from the flank of the recipient, taking care that the excision was only superficial, not exposing the body cavity of the embryo to the external medium. The donor animal cap was cut in half and placed into the recipient at the excision site. The recipient embryos were then carefully placed in agarose molds submerged in 0.1× MMR with nicotine (0.1 mg/ml). The molds hold the graft in place and allow the embryo to heal. Embryos were allowed to heal for 4–8 h, then washed and placed in fresh 0.1× MMR with nicotine at 14°C overnight. Embryos were then cultured at 18°C until analyzed.

### Beta-Galactosidase Enzymatic Detection

Animals injected with β-galactosidase (β-gal) mRNA were fixed (30 min in MEMFA at RT) at the relevant stages, washed twice in PBS with 2 mM MgCl_2_, and stained with X-gal (Roche Applied Sciences, Indianapolis, IN, USA) staining solution at 37°C for 3 h. Tadpoles were then rinsed three times in PBS followed by dehydration through sequential in incubation in 25%, 50%, 75%, and 100% methanol. Tadpoles were then incubated in 30% H_2_O_2_ in methanol overnight for bleaching. Tadpoles were then washed in 100% methanol sequentially rehydrated to PBS and imaged.

### Histology

Agarose sectioning was performed as previously described (Blackiston et al., 2010b). Briefly, stage 45 tadpoles were fixed for 2 days in MEMFA (Sive et al., 2000), washed in PBT, dehydrated in methanol, rehydrated back in PBT, embedded in 4% agarose and sectioned at 100 μm using a Leica vibratome, followed by hematoxylin and eosin staining.

### Statistics

Statistical analyses were performed using GraphPad Prism7. At least three independent experiments were conducted with *N* > 50 embryos for each treatment group, with embryos collected from multiple animals across independent clutches. Data were analyzed by *t*-test (for two groups) or ANOVA (for more than two groups, with Tukey’s multiple comparison test) as indicated with each experiment.

### Data Availability

All data generated or analyzed during this study are included in this article and Supplementary Material is available from the corresponding author upon request.

## Results

### Overexpression of HCN2 in Both Local (Neural) and Distant (Ventral) Tissue Rescues/Protects Nicotine Exposure-Induced Brain Defects

To test whether HCN2 expression in local or non-local tissues can rescue nicotine-induced brain defects, we injected *Hcn2-WT* mRNA into precursors of the brain [dorsal blastomeres (Moody, 2018)] or ventral tissues (ventral blastomeres) at four-cell stage followed by exposure of *Xenopus* embryos to nicotine (Pai et al., 2018). Untreated, uninjected embryos and untreated, *Hcn2-DN (Dominant Negative)* mRNA-injected embryos were used as controls. We injected mRNA at the four-cell stage, using the fate map distinguishing dorsal from ventral precursors to establish the first proof-of-principle of long-range activity. To optimize the likelihood of efficient rescue, we sought to target either the whole neural plate region or equivalently a large portion of the non-neural region to achieve the maximum demonstration of long-range repair. Co-injection of lineage tracer *β-galactosidase* mRNA validated our targeting of neural and non-neural regions (Figures 1A–D). Brain morphology was evaluated at stage 45 (Figures 1E–Q). Tadpoles from control embryos exhibited correctly patterned (Pratt and Khakhalin, 2013) brain tissue (Figures 1E,K,N,Q). Nicotine exposure caused an increased incidence of abnormal brain morphology (59% tadpoles with brain defects) in comparison to controls (7% tadpoles with brain defects; Figures 1F,L,O,Q). Phenotypes included the absence of nostrils, absence of forebrain, absence of forebrain and midbrain, truncated and mispatterned nostrils, forebrain, and midbrain. Eye development was also affected, resulting in absent or incompletely formed eyes (Pai et al., 2012a). The average distribution of these phenotypes in nicotine-exposed tadpoles is listed in Supplementary Table S1. In contrast, embryos with dorsal blastomere (neural) *Hcn2-WT* mRNA injection showed significant rescue or protection of nicotine-induced abnormal brain morphology (27% tadpoles with brain defects), with a large scale brain patterning similar to that of controls (Figures 1H,Q). Embryos with only dorsal *Hcn2-WT* mRNA injection (no nicotine exposure) were not significantly different from the controls (5% tadpoles with brain defects; Figures 1G,Q). Thus, overexpression of HCN2-WT channels in neural tissue is able to significantly counteract the teratogenic effects of nicotine exposure on brain patterning.

Remarkably, embryos with *Hcn2-WT* mRNA injected into ventral blastomeres (non-neural precursors, distant from the brain) also showed a near-complete rescue/protection of brain morphology (16% tadpoles with brain defects) with large-scale brain patterning similar to controls (Figures 1J,M,P,Q). Embryos with only ventral blastomere *Hcn2-WT* mRNA injection (no nicotine exposure) were not significantly different from the controls (6% tadpoles with brain defects; Figures 1I,Q), confirming that HCN2-WT expression in ventral tissues does not harm normal development. Thus, overexpression of HCN2-WT channels in non-CNS tissues can significantly counteract the teratogenic effects of nicotine exposure on brain patterning.

To quantify brain morphology, we used geometric morphometrics at stage 45 (Webster and Sheets, 2010; Figure 2). Landmarks were chosen based on transition points between different brain regions and lateral and anterior outermost points of the head (Figure 2A) and recorded for tadpoles for each of the controls, nicotine, nicotine + dorsal (local/within neural tissue) *Hcn2-WT* mRNA, and nicotine + ventral (non-local/within non-neural tissue) *Hcn2-WT* mRNA. Canonical variate analyses with Procrustes distances between each of the groups quantitatively revealed changes in the length of the brain relative to head shape between treatment conditions (Figure 2B) and the direction of changes in shape (Figures 2C,D). Control and nicotine + dorsal-HCN2-WT embryos were not significantly different in shape, but both were significantly different from nicotine-exposed embryos (Figure 2B). Also, control and nicotine + ventral-HCN2-WT embryos were not significantly different in shape (Figure 2B), but both were significantly different from nicotine-exposed embryos. Thus, both dorsal (local, within neural tissue) or ventral (non-local, in non-neural tissue) *Hcn2-WT* mRNA microinjections protect or rescue brain morphology to the wild-type state despite nicotine exposure.

### Both Local (Neural) and Distant (Ventral) HCN2 Restores Normal Expression of Neural Marker Genes

To gain insight into the molecular mechanisms and determine whether local (neural) and distant (ventral) HCN2 channel interventions affect the known brain patterning transcriptional regulators, we analyzed the expression of canonical factors *otx2* (forebrain and midbrain; Acampora et al., 1995) and *xbf1* (forebrain; Bourguignon et al., 1998). Embryos were analyzed at stage 25 by *in situ* hybridization (Figure 3). Nicotine-exposed embryos showed significantly reduced expression of *otx2* (both in area and intensity; Figure 3C) and reduced or mispatterned *xbf1* expression (Figure 3D) compared to controls (uninjected and untreated embryos; Figures 3I,J). Crucially, both dorsal blastomere (local/neural) and ventral blastomere (distant) *Hcn2-WT* mRNA-injected embryos showed normal *otx2* and *xbf1* expression (Figures 3E–J). Thus, both neural and distal HCN2 channel misexpression induces correction of *otx2* and *xbf1* expression despite nicotine exposure, suggesting that the action of the bioelectric repair functions upstream of these early brain patterning genes.

### Overexpression of HCN2 in Both Local (Neural) and Distant (Ventral) Tissue Restores Normal Membrane Voltage Prepattern of Nicotine-Treated Embryos

To elucidate the biophysical mechanism by which HCN2 overexpression induced corrective changes in brain gene expression, we characterized the effect of nicotine and *Hcn2* microinjections on the endogenous bioelectric prepattern (distribution of resting potentials) known to be a critical regulator of the neural transcription factors and brain patterning (Pai et al., 2015b). We evaluated embryos between stages 15–17 using a combination of *in vivo* imaging, voltage reporter dyes (Adams and Levin, 2012), and whole-cell membrane voltage recordings (Pai et al., 2015b; Figure 4). Whole-cell electrophysiological recordings of membrane voltage from neural plate cells and flanking ectodermal cells were used as calibration points for the voltage reporter dye images, and the fluorescence intensities were analyzed against these calibration points to approximate membrane voltages at different points within the developing embryos (Figure 4).

Nicotine-treated embryos showed a significantly depolarized neural resting membrane potential (depolarized by ~30 mV) in comparison to controls (uninjected and untreated embryos; Figures 4A,B,G,H). *Hcn2-WT* mRNA injected embryos (either dorsal or ventral microinjections) were not significantly different from the controls (Figures 4A,C,E,G,H). Interestingly, nicotine-exposed embryos that were injected with *Hcn2-WT* mRNA, either dorsally or ventrally, both showed restored neural plate hyperpolarization in comparison to nicotine-only treated embryos (Figures 4A,B,D,F,G,H). Thus, both local (neural) and distant (ventral) HCN2 channel expression restores the correct neural membrane voltage prepattern despite the presence of nicotine. We conclude that bioelectric changes induced in a distant region of the embryo can affect the bioelectric prepattern of the nascent brain and that ventral HCN2 injections are inducing repair by correcting the nicotine-mediated disruption of the neural plate bioelectric prepattern.

### Model of Distant HCN2-Mediated Correction of Membrane Voltage Prepattern Disruption

How does altering the electrophysiological properties of distant cells (HCN2 misexpression in ventral regions) correct the membrane voltage prepattern in the anterior neural plate? Cell-cell connections across tissues can drive highly complex, non-intuitive, spatiotemporal changes in membrane voltage patterns that are best understood *via* simulations (Cervera et al., 2015, 2016a,b, 2018; Pietak and Levin, 2016, 2017; Brodsky and Levin, 2018). Hence, we formulated a minimal computational physiological model of spatial membrane voltage patterns to understand long-range rescue/protection by distant ion channel misexpression (Supplementary Material and Figure 5). This model incorporated both, past results (Pai et al., 2015a,b, 2018) and the novel data reported here (Figures 1–4). This model focused specifically on the membrane voltage dynamics, not restricting absolute tissue size or timescale: the objective was specifically to understand non-cell-autonomous membrane voltage propagation. We then extracted from this model specific predictions for experimental membrane voltage perturbations that should or should not be successful in reversing or preventing nicotine-induced brain defects.

The key experimental results on which this model is formulated are as follows. Beginning neurulation the neural plate cells are significantly hyperpolarized in comparison to the surrounding depolarized ectodermal cells (Pai et al., 2015a,b, 2018). We found that it is not the individual ion fluxes (such as Na^+^ or K^+^ flux) but the membrane voltage of these cells that regulate embryonic patterning (Pai et al., 2012a, 2015b). In the case of brain patterning, we found that it is not the absolute values of membrane voltage but the contrast/difference in membrane voltage pattern between neural plate and ectoderm that is crucial for proper brain patterning (Pai et al., 2015b). Eliminating this contrast/difference results in serious brain defects irrespective of the absolute membrane voltage of any given tissue. These membrane voltage patterns act locally and over long-range to control key cell behaviors such as proliferation and apoptosis during brain patterning (Pai et al., 2015a,b). Also, these membrane voltage signals are transduced through GJs and regulate canonical gene regulatory networks and biochemical signals orchestrating brain patterning (Pai et al., 2015b). Lastly, these membrane voltage patterns are altered during many developmental brain deformities and these deformities can be corrected by precise modulation of these membrane voltage patterns (Pai et al., 2015b, 2018).

In our model (Supplementary Material), complex developmental membrane voltage patterning is depicted at the multicellular level in cell ensembles. We used an equivalent circuit strategy, sufficient to qualitatively reproduce the observed behavior of the importance of contrast (difference) of membrane voltage patterns during brain patterning as described above and shown in the results of this manuscript (Pai et al., 2015b, 2018; and Figures 1–4). Since individual ion fluxes are important due to their contribution to overall V_mem_, a single cell’s membrane voltage is represented as controlled by two counteracting voltage-gated ion channel aggregates of maximum conductances G_pol_ and G_dep._ G_pol_ is regarded as an *effective sum* of channels that promote the polarized (*pol*) cell state while G_dep_ is regarded as an *effective sum* of channels that promote the depolarized (*dep*) state. A key characteristic of our approach is that the single-cell* state* can be modulated at the ensemble level because of the coupling of a given cell with the neighboring cells (Cervera et al., 2016b, 2018, 2019). This coupling is allowed by the intercellular gap junction conductance *G*_ij_ that permits the transfer of ionic currents and signaling molecules between two adjacent cells (Cervera et al., 2016b, 2018, 2019). Such gap-junction connections between embryonic cells are crucial for proper embryonic tissue patterning (Spray et al., 1981; Warner, 1985; Pai et al., 2015b; Mathews and Levin, 2017).

In the multicellular ensemble, the membrane voltage states are modulated by: (1) the relative values of the single-cell channel conductances G_pol_ and G_dep_ promoting the polarized and depolarized cell states, respectively; (2) the relative values of the maximum intercellular GJ conductance G^0^ and the single-cell conductances of the connected cells; (3) the relative size and proximity of the polarized dorsal region (representing the polarized neural tube) and the polarized tissue patch (representing the HCN2-expressing non-neural tissue) that is created within the depolarized non-neural region. A limitation of our approach is that external actions should also produce additional diffusion-reaction processes characterized by experimental transient times much higher than those obtained with purely electrical mechanisms, as previously explained (Cervera et al., 2016b, 2018). The results of Figure 5 concern only the steady-state system states. Besides, the particular current-voltage curves of all ion channels and gap-junctions used are also shown in the respective Supplementary Figures S1–S3 of Supplementary Material.

Our goal was not to construct a maximally-realistic simulation of the *Xenopus* neural tube but to identify the key drivers of this developmental physiology and understand whether and how HCN channels can underlie the propagation of resting potential changes over long distances in developing tissue. The concerted action of the HCN2 channels is simulated by an effective inward-rectifying conductance which opens to cations at hyperpolarized potentials (Biel et al., 2009; Benarroch, 2013). For strongly depolarized cells, however, this channel closes giving an almost zero residual conductance. However, given the ionic gradients in *Xenopus* cells (high intracellular Na^+^ and K^+^ (Gillespie, 1983) in comparison to external media), the resulting effective channel acts to polarize rather than to depolarize the *Xenopus* cells (Pai et al., 2018). We qualitatively described the membrane voltage states of the cells in two generic dorsal (neural) and ventral (non-neural) regions within this multicellular ensemble.

The control case (Figure 5A) represents the endogenous physiological condition. The neural region is marked by the circle and the rest represents the non-neural region of the embryo. Differences in the baseline levels of the G_pol_ and G_dep_ conductances of the cells within these regions result in a hyperpolarized neural plate region (−55 mV) in comparison to the rest of the embryo (−5 mV; Supplementary Material). This pattern is consistent with the observed membrane voltage patterns in *Xenopus* embryos (Figure 4 and in Pai et al., 2015b). Figure 5B corresponds to the depolarization of the neural plate due to nicotine-exposure. The depolarizing effect of nicotine is simulated by lowering the conductance G_pol_ in the neural region, which prevents this neural region from reaching the polarized state of Figure 5A (Pai et al., 2015b; Supplementary Material). The nicotine-exposed multicellular ensemble is then used to test the effect of externally introducing the HCN2 channel in the patch of the non-neural region.

The HCN2 channel misexpression in the non-neural tissue was simulated by increasing the context-specific conductance G_pol_ in the small patch of tissue (Figures 5C–F). Remarkably, the ectopic hyperpolarized patch could repolarize the neural region at a distance (Figure 5C). However, this neural repolarization did not occur if the polarized patch was too small (Figures 5C,D) or was placed too far away (Figures 5E,F). Thus, three key predictions arise from the simulations: (1) a patch of HCN2-expressing cells in the non-neural tissue should be able to restore the membrane voltage patterns in the neural plate of nicotine-exposed *Xenopus* embryos; (2) if the patch of HCN2-expressing cells is small, it should fail to rescue membrane voltage patterns in this neural plate of nicotine-exposed *Xenopus* embryos; and (3) the patch of HCN2-expressing cells needs to be sufficiently close to the neural plate to rescue the membrane voltage patterns in the nicotine-exposed *Xenopus* embryos. This model reveals the minimal aspects necessary for long-range bioelectric influence propagation; it explains the ability of HCN2 expression in remote regions to influence the membrane voltage of anterior neural tissues (Figure 4).

### Ectopic HCN2-Expressing Transplants Induce Repair of Nicotine-Induced Defects

To test the model’s predictions and to determine whether HCN2’s repairing activity could operate if it was not present during the very early stages of development, we developed a tissue transplant assay (Figure 6A). We microinjected either *Hcn2-WT* mRNA or *Hcn2-DN* (dominant-negative) mRNA, with a fluorescent lineage tracer *Tomato* mRNA (Figure 6A, red), into donor embryos at the one-cell stage. The animal caps from donor embryos were excised and transplanted into the flank of age-matched (stage 13) nicotine-exposed host embryos (see methods for details). The embryos were allowed to heal and develop to stage 45, and brain morphology was evaluated (Figures 6B–J). This transplant assay allowed us to cleanly test the effects of an ectopic patch of HCN2-expressing cells at a well-defined point in developmental time and location.

Control (uninjected, untreated, un-transplanted) tadpoles had correctly patterned brain tissue (Pratt and Khakhalin, 2013), with well-formed nostrils, olfactory bulbs/forebrain, midbrain, and hindbrain (Figure 6B). Nicotine exposure induced a significant increase in the incidence of brain defects (58% tadpoles with brain defects) in comparison to controls (5% tadpoles with brain defects; Figures 6C,H). Strikingly, nicotine-exposed embryos with *Hcn2-WT*-expressing transplants (appearing in the somatic ventral region; Figure 6E) showed a significant rescue of brain defects (22% tadpoles with brain defects; Figures 6D,E,H). In contrast, the nicotine-exposed embryos with *Hcn2-DN-* or *tomato*-only expressing transplants caused no rescue (65% and 59% tadpoles with brain defects, respectively; Figures 6F–H). We conclude that HCN2-WT channel-expressing transplanted donor tissue induces repair of brain morphology defects at a distance and that this rescue is still effective when begun at stage 13. This result also validates the model’s prediction that HCN2-expressing cells in non-neural tissues should be able to rescue nicotine-induced abnormal brain morphology.

Next, we sorted the nicotine-exposed tadpoles that received *Hcn2-WT+tomato* mRNA transplants based on the size of the red fluorescent tomato patch and assessed the degree of rescue. Nicotine-exposed embryos that received large transplants showed a significantly better rescue (27% tadpoles with brain defects) than the nicotine-exposed embryos that received small transplants (83% tadpoles with brain defects; Figure 6I). This result validates the theoretical prediction that larger patches of HCN2-expressing cells in non-neural tissues should rescue nicotine-induced brain defects better than smaller patches of HCN2-expressing cells.

Analogously, we sorted the nicotine-exposed tadpoles that received *Hcn2-WT* +*tomato* mRNA tissue transplants based on the location of transplant (either in the fin tissue far away from CNS or in tissues close to CNS). Interestingly, nicotine-exposed embryos that received transplants near the CNS showed better rescue (0% tadpoles with brain defects) than those that received transplants in the fin region (81% tadpoles with brain defects; Figure 6J). This result agrees with the prediction that a patch of HCN2-expressing cells closer to the neural tissue should rescue nicotine-induced brain defects better than the patch of HCN2-expressing cells that are farther away from the neural tissue.

### Endogenous HCN2 Channel Activators Rescue Nicotine-Induced Brain Defects Even After Delayed Treatment

The induction of improvements in brain morphogenesis by transplants occurring well after teratogen exposure revealed that the effect is not simply prevention but also repair. To confirm this and establish a strategy that does not require tissue transplants, we next turned to human-approved ion channel activating drugs. To discover whether small molecule activation of endogenous HCN2 channels would be sufficient to rescue nicotine-induced brain defects, we used two well-known HCN channel activators: lamotrigine (LT) and gabapentin (GP; Poolos et al., 2002; Surges et al., 2003; Postea and Biel, 2011; Brennan et al., 2016). While these drugs may have additional targets besides HCN, we used these reagents not to prove the involvement of HCN2 (which we have already shown using highly specific molecular-genetic reagents; Figures 1, 6; Pai et al., 2018), but to determine whether a small molecule agent that activates native HCN2 channels at later stages is sufficient to overcome teratogen exposure. Much higher concentrations of these drugs than the ones used in this study could affect development adversely. However, we found and used a non-toxic dose of these drugs where the main effect is an improvement of developmental outcomes.

*Xenopus* embryos were exposed to nicotine (stages 10–35), and then either concurrently (stage 10–35) or after a delay (stage 25–35), treated with lamotrigine or gabapentin. Untreated embryos and only lamotrigine- or gabapentin-treated embryos served as respective controls. The embryos were allowed to develop to stage 45, and brain morphology was evaluated (Figure 7). Control tadpoles had correctly patterned brain tissue, with well-formed nostrils, olfactory bulbs/forebrain, midbrain, and hindbrain (Figure 7A), while nicotine exposure caused brain defects (67% and 68% tadpoles with brain defects, respectively) in comparison to controls (5% and 8% tadpoles with brain defects, respectively; Figures 7B,G,H). Concurrent lamotrigine or gabapentin treatment showed a significant rescue (15% and 23% tadpoles with brain defects, respectively) of nicotine-induced defects (Figures 7D–H). Similarly, delayed treatment with lamotrigine and gabapentin showed significant rescue (only 10% and 16% tadpoles with brain defects, respectively) of the brain defects (Figures 7G,H). Lamotrigine- and gabapentin-only treated tadpoles showed no difference in brain morphology (2% and 11% tadpoles with brain defects, respectively) compared to controls (5% and 8% tadpoles with brain defects, respectively; Figures 7A,C,E,G,H), showing that these drugs alone do not impair brain development.

To quantify brain morphology, we used geometric morphometrics (Webster and Sheets, 2010; Figure 8). Landmarks were chosen based on transition points between different brain regions and lateral and anterior outermost points of the head at stage 45 (Figure 8A), and measurements were recorded for *N* > 10 tadpoles for each of the following conditions: controls, nicotine, nicotine+lamotrigine, and nicotine+gabapentin. Canonical variate analyses (Figure 8B) were run on the data set, with Procrustes distances measured between each of the groups quantitatively revealing changes in the length of the brain relative to the head shape across treatment conditions (Figures 8B–D). Control, nicotine+lamotrigine, and nicotine+gabapentin embryos were not significantly different in shape (Figure 8B, orange, blue, and cyan ellipses), but were significantly different from nicotine-exposed embryos (magenta ellipse). Thus, quantitative shape analysis reveals that the treatment of nicotine-exposed embryos with lamotrigine or gabapentin well after initial teratogen exposure rescues nicotine-induced brain defects to near wild-type.

### Lamotrigine and Gabapentin Treatments Restore Normal Membrane Voltage Prepattern of the Nascent Brain in Nicotine-Treated Embryos

To determine how lamotrigine or gabapentin rescue brain defects, we next tested the hypothesis that they restore the endogenous neural plate membrane voltage prepattern required for brain patterning (Pai et al., 2015b). by evaluating resting potential distributions in embryos treated from stage 10 (Figure 9). Nicotine-treated embryos showed a significantly depolarized potential (by ~28 mV) vs. controls (Figures 9A,B,E,F). In contrast, nicotine-exposed embryos treated with lamotrigine or gabapentin showed a significantly hyperpolarized neural plate (by ~33 mV) compared to controls (Figures 9C–F). Thus, both lamotrigine and gabapentin hyperpolarize the neural plate membrane voltage prepattern despite the presence of nicotine, revealing that these compounds repair brain morphology in the same way as does HCN2 overexpression.

### Lamotrigine and Gabapentin Restore Associative Learning in Nicotine-Exposed Tadpoles

**Figure 10:**
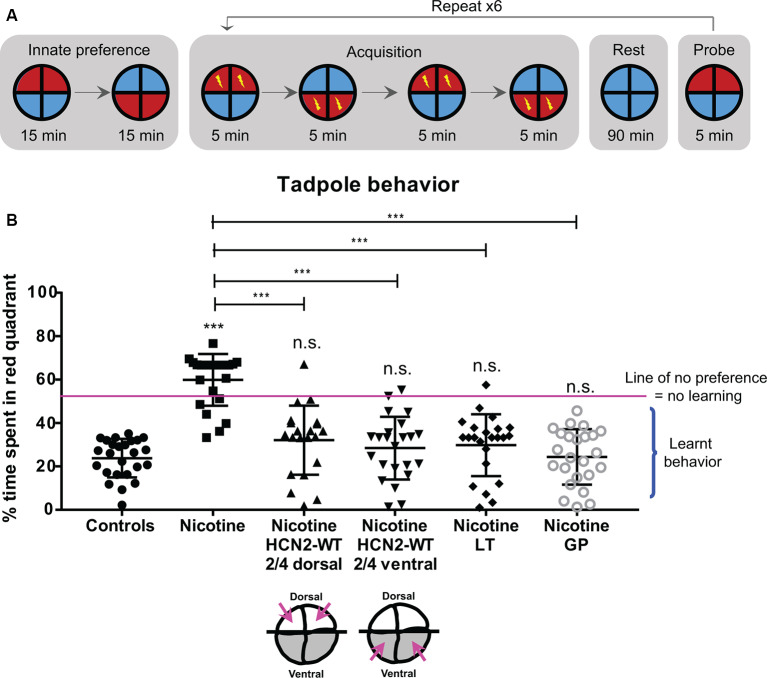
HCN2 channels (local and distant) and HCN2 channel agonists restore associative learning capacity in nicotine-exposed embryos. Associative learning analysis for stage 45–50 tadpoles with indicated treatments. Lamotrigine (LT, stages 10–35) or gabapentin (GP, stages 10–35). **(A)** The training regime in the behavior analysis machine consisting of an innate preference test, a training phase (acquisition), a rest period, and a learning probe. Automated software executed a training cycle where animals received a shock when occupying the red half of the arena. Training, rest, and testing sessions were repeated a total of six times across the trial. **(B)** Quantification of time spent by each tadpole in the red-lit area during the final testing probe for each of the indicated treatments. Percentage of time spent by each tadpole in red light for each experimental group are Controls—24%, Nicotine—60%, Nicotine+HCN2-WT 2/4 dorsal—32%, Nicotine+HCN2-WT 2/4 ventral—27%, Nicotine+LT—28%, and Nicotine+GP—24%. Data represented as mean ± SD, ***p < 0.001, n.s.: non-significant (one-way ANOVA with Tukey’s *post hoc* test for *N* > 20 tadpoles for each treatment group).

We next assayed the prospects for the functional rescue of behavioral performance. Using an automated behavior analysis platform (Blackiston et al., 2010a; Figure 10A), a tadpole can be trained to avoid a moving red light, enabling quantification of cognitive performance (Figure 10B). A tadpole was classified as “having learned” if its preference for red light dropped below 40% of time spent in red light, averaged across the final three probe sessions of the experiment. This approach has been successfully used to quantitatively evaluate brain function and learning performance under surgical, pharmacological, and genetic manipulations (Blackiston and Levin, 2013a,b; Blackiston et al., 2017).

Forty percent of nicotine-exposed animals showed no obvious motor problems or movement abnormalities, and only these were tested in the learning assay. Morphologically normal nicotine-exposed tadpoles failed to learn to avoid red light with overall ~60% of the time spent in red light (Figure 10B). In comparison, control tadpoles learned to avoid red light with overall only ~24% of time spent in red light (Figure 10B). However, nicotine-exposed embryos receiving dorsal (local/neural) or ventral (distant/non-neural) *Hcn2-WT* mRNA at the four-cell stage showed significant improvement in learning ability with overall only ~32% and ~27%, respectively, of time spent in red light (Figure 10B). Similarly, nicotine-exposed embryos treated with lamotrigine (stage 10–35) or gabapentin (stage 10–35) also showed significant improvement in learning ability with overall only ~28% and ~24%, respectively, of time spent in red light (Figure 10B). Thus, both local (neural) and distant (non-neural) overexpression of HCN2 channels, as well as drug-based activation of native channels, restore cognitive learning abilities in nicotine-exposed tadpoles.

## Discussion

A specific spatial distribution of resting potential is an endogenous prepattern necessary for normal brain development; moreover, externally enforcing this pattern restores brain pattern despite a range of genetic, chemical, and mechanical insults (Pai et al., 2015a,b, 2018; Herrera-Rincon et al., 2017). Changes in bioelectric state are transduced by gap-junctions and calcium flux and regulate key brain patterning transcription factors (Pai et al., 2015a,b). Previously we showed that the classic neuroteratogen nicotine (Slotkin et al., 2005; Huizink and Mulder, 2006; Slotkin, 2008; Velazquez-Ulloa, 2017) induced brain morphology defects by disrupting these bioelectric prepatterns and presented a detailed model of brain cell physiology and downstream gene expression changes (Pai et al., 2018). Modulating these bioelectric patterns using HCN2 channel overexpression revealed the predicted amelioration of nicotine-induced brain defects. Overexpression of the HCN2 channel itself does not cause any phenotypic defects but instead corrects mild brain deformities seen in control embryos (Pai et al., 2018). This effect of HCN2 is mainly due to their context-specific action, where the channels open only under hyperpolarizing conditions (−40 mV to −70 mV; neural plate) and remain closed in depolarized cells (non-neural ectoderm). Thus, HCN2 channels magnify the voltage differential between two cell fields (neural and non-neural). In areas where these differences are already strong (normal embryos) they have little effect because compartment boundaries are already set and the apposition of their distinct V_mem_ already activates downstream mechanisms. However, in regions where boundaries have become significantly weakened (e.g., because of teratogens like nicotine), HCN2 increases the differential to normal levels, correcting morphogenesis (Pai et al., 2018).

Here, we sought additional insight into endogenous bioelectric controls of brain development and established a proof-of-principle for regenerative medicine approaches, by tackling two key questions that remained open: (1) can patterning be controlled by bioelectric states of distant tissues; and (2) can a repair be induced by targeting native channels instead of over-expressing native HCN2. First, by targeting HCN2 mRNA to dorsal or ventral tissues, followed by marker analysis, physiological profiling, and morphometrics, we found that either local or long-range HCN2 overexpression can restore the normal voltage pattern, the expression of classic neural transcription factors *otx*2 a*nd xbf1*, and brain morphology—all of which are otherwise disrupted by nicotine exposure (Figures 3, 4). Our marker analysis revealed the bioelectric influence to operate upstream of the known regulators of brain size and shape (*Otx2, Xbf1*), likely using the same transduction mechanisms we previously identified (Pai et al., 2015a,b, 2018). In the future, high-resolution analysis of HCN2 expression in individual blastomeres of 16 and 32 cell embryos could identify the smallest regions that are still sufficient for inducing repair.

How does distant (outside neural plate/non-CNS) HCN2 overexpression in nicotine-exposed embryos restore the membrane voltage pattern? As with many gene-regulatory circuits, membrane voltage control involves many complex (positive and negative) feedback loops, making it difficult to directly predict the time evolution of multicellular tissue (system-level) distributions of resting potentials (Adams and Levin, 2013; Levin and Martyniuk, 2018); thus, computational modeling is helpful to understand the dynamics and possible behaviors of bioelectric tissues (Cervera et al., 2018; Pietak and Levin, 2018). We constructed a simulation to map spatial membrane voltage changes to analyze and understand the observed experimental results (Supplementary Material; Cervera et al., 2016b; Cervera et al., 2018).

The distant HCN2 overexpression was simulated as a patch of polarized cells in the non-neural region of nicotine-exposed embryos. This polarized patch of cells repolarizes the neural region in nicotine-exposed embryos *via* the known presence of intercellular gap-junction connections between the polarized patch and the neural region (Figure 5). The patch polarization spreads mainly in the direction of the neural region because the bulk of the region ventral to the patch is depolarized and dynamically buffers against the spread of polarization in this region. A patch of sufficient size spreads polarization into the neural region, while small HCN2-expressing polarized patches fail to restore the neural membrane voltage (Figure 5). The plasticity of *Xenopus* embryonic development allowed us to test the model’s predictions using embryonic transplant experiments (Figure 6). An ectopic patch of HCN2 tissue transplanted onto nicotine-exposed embryos was sufficient to rescue the brain morphology, definitively confirming the non-cell-autonomous nature of the effect as well as other predictions of the model concerning size and distance of the patch. Our model explains the minimal dynamics necessary for the highly surprising finding that introducing a hyperpolarizing region on an embryo enables control of membrane voltage states (and downstream morphogenesis) in a different region.

Certainly, we do not rule out the possibility of additional biochemical, mechanical, or other signals such as exosomal communication (Danilchik and Tumarkin, 2017), electrically coupled nanotubes (Wang et al., 2010), and/or cytonemes (Danilchik et al., 2013; Kornberg and Roy, 2014) that might propagate the effects of distant membrane voltage changes into downstream effects *in vivo*. Because this simple equivalent circuit model is compatible with a variety of biological contexts (underlying channel profiles), such long-range propagating influences may be sought in other cases, for example in the propagation of bioelectric states during limb injury (Busse et al., 2018) or cancer (Chernet and Levin, 2014; Chernet et al., 2015). Moreover, it provides the minimal necessary condition to recapitulate such tissue propagation signals in synthetic bioengineering contexts (McNamara et al., 2016, 2018). Using the insights of such models, future efforts will focus on developing stimulation protocols to maximize the distance at which reparative effects can be induced in a target region *in vivo*.

Our data show that distant membrane voltage states can rescue brain patterning as long as they are close to neural tissue and are significantly large. These signals can regulate the transcription of key genes, likely due to small molecules or electric current propagating through gap junctions during this long-range communication. One of the remaining open questions concerns why some cells along with the path act as mere relays of information while other cells interpret this information and act because of their different transcriptional states. This is currently a major area of investigation, using synthetic minimal bioelectric tissues to understand how voltage boundaries form and how voltage differences are transduced to regulate downstream transcriptional responses (Cervera et al., 2018, 2016b; McNamara et al., 2018, 2020).

To explore the possibility that systemic, human-safe bioelectric drugs can be exploited for new strategies in regenerative medicine, we utilized two FDA-approved molecular activators of HCN channels: lamotrigine (Goldsmith et al., 2003; Postea and Biel, 2011) and gabapentin (Surges et al., 2003; Wiffen et al., 2017), both of which are in wide use in patients (Dolk et al., 2016). While these drugs may have additional targets besides HCN, our molecularly-specific experiments with HCN2 mRNA demonstrate that the HCN2 activation activity is sufficient to induce repair. While high doses of these compounds might affect development on their own, we show that it is possible to identify a dose where the main effect is to strengthen membrane voltage boundaries and rescue complex organogenesis. Both lamotrigine and gabapentin successfully rescued brain defects in nicotine-exposed embryos (Figures 7–10). Importantly, we did not have to precisely target the drug exposure to a specific region or tissue of the embryo. Soaking the entire embryo in drug-containing media resulted in localized effects without requiring localized exposure, due to the sensitivity of HCN2 to local voltage conditions—it is a context-sensitive reagent having different effects on the membrane voltage of cells in the middle vs. surrounding of the neural tube.

It is possible that lamotrigine and gabapentin might also affect other HCN channels or indeed other ion translocators (e.g., inhibitors of Na^+^ and Ca^2+^ channels; Goldsmith et al., 2003; Eroglu et al., 2009); however, our data (Figures 1–6, Pai et al., 2018) show that HCN2 is sufficient to induce brain repair, while the drugs do not induce any defects on their own. Also, given the ionic gradients in *Xenopus* cells (high intracellular Na^+^ and Ca^2+^ (Gillespie, 1983) in comparison to external media), Na^+^ and Ca^2+^ channel opening would cause hyperpolarization, and hence the non-HCN action of drug (inhibition of Na^+^ and Ca^2+^ channels) would actually prevent the hyperpolarization we observed, further pointing towards HCN2 mediated action of these drugs. As numerous ion channel blockers and openers are already approved for human use for arrhythmias, epilepsy, etc., future efforts at identifying novel and more specific HCN2 drugs may be warranted, as part of a strategy to identify useful electroceuticals (Churchill et al., 2019).

**Figure 11:**
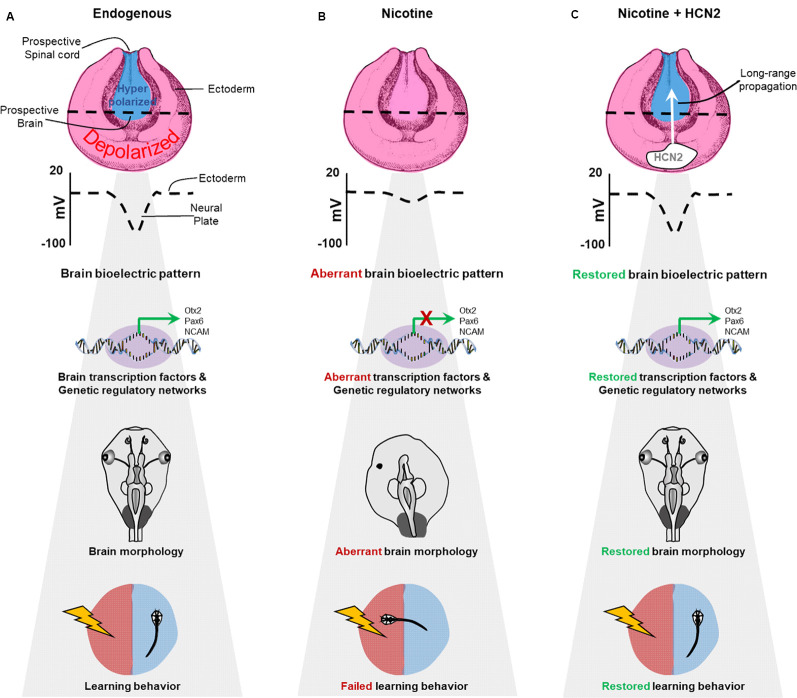
Schematic model of bioelectric signaling in brain teratogenesis and long-range repair. **(A)** Our new data and those of previous studies (Pai et al., 2015b, 2018) show that the membrane voltage difference between the hyperpolarized neural plate and depolarized surrounding ectoderm is crucial for correct gene expression pattern, proper brain morphology, and normal learning behavior. **(B)** Embryonic nicotine exposure erases this crucial membrane voltage difference and leads to aberrant gene expression patterns, brain morphology defects, and impaired learning behavior. **(C)** Transplanting HCN2 channel tissue onto nicotine-exposed embryos is sufficient to restore the membrane voltage difference over long-range and rescue gene expression patterns, brain morphology defects and learning behavior in these animals.

Two other aspects should be noted. First, although the nicotine exposure started at stage 10, reparative drug treatment could be initiated after a significant delay (stage 25). We do not know the limit of the delay, but the ability to repair defects significantly later than teratogen exposure began is an attractive feature of this strategy. Second, the repair was not merely morphological but also functional. Remarkably, complex learning behavior can be restored by simple drug treatment, channel misexpression, or implant of HCN2-expressing cells, not requiring high-resolution spatiotemporally patterned manipulation (Figure 10). Thus, a relatively simple bioelectric prepattern can initiate a complex downstream program of gene expression, tissue morphogenesis, and learning behavior (Figure 11). The ability to rescue the function of such a complex, integrated organ, despite the presence of a potent teratogen, underscores the power of capitalizing on biophysical controls of developmental modules. Strategies taking advantage of bioelectric models of health and disease states, as well as the plethora of well-characterized ion channel drugs, could offer a new way to trigger complex patterning modules for applications in birth defects, regenerative medicine, cancer, and bioengineering.

## Data Availability Statement

All datasets generated for this study are included in the article/Supplementary Material.

## Ethics Statement

All experiments were approved by the Tufts University Animal Research Committee (M2017-53) in accordance with the guide for care and use of laboratory animals.

## Author Contributions

VP performed experiments. VP and ML designed the experiments and interpreted the data. VW assisted with the geometric morphometric analysis and *in situ* hybridization assays. EL assisted in membrane voltage dye analysis. JC and SM designed and performed the simulations. VP and ML wrote the manuscript together.

## Conflict of Interest

The authors declare that the research was conducted in the absence of any commercial or financial relationships that could be construed as a potential conflict of interest.

## Supplementary Material

FIGURE S1 The ion channel current (*I*)–voltage (*V*) curves show the outward and inward-rectifier currents and the total current (central curve) as a function of the voltage *V* for two effective channels (*G*_ref_ = 1 nS; Cervera et al., 2014; Law and Levin, 2015; Cervera et al., 2018). **(A)** Two counteracting voltage gated channels and **(B)** a leakage channel counteracting a voltage-gated channel. The two stable (*outer filled circles*) and the unstable (*central hollow circle*) *membrane potentials* shown are particular values of the voltage *V* obtained from the condition of zero current, *V*_mem_ = *V*(*I* = 0). The stable values approximately correspond to the polarized and depolarized potentials assumed in these curves. In this model, the inward-rectifying channel of conductance *G*_pol_ acts to fix the polarized potential *E*_pol_ while the outward-rectifying channel **(A)** or the leakage channel **(B)** of conductance *G*_dep_ favors the depolarized potential* E*_dep_, according to the approximate equation *V*_mem_ = (*G*_pol_*E*_pol_ + G_dep_*E*_dep_)/(*G*_pol_ + *G*_dep_); see Cervera et al. (2014); Law and Levin (2015); Cervera et al. (2018) and references therein for details.

FIGURE S2 The voltage-gated conductances as a function of the cell potential (voltage) *V*.

FIGURE S3 Experimentally, the gap junction conductance *G_ij_*/*G*_ref_ between two neighboring cells *i* and *j* follows a bell-shaped function of the difference *V_i_* –* V_j_* [*left*, **(A,C)**] which confers the intercellular plasticity [*right*, **(B,D)**] of the effective nodes in the multicellular ensemble. The curves show two high and low values of the maximum conductance *G*^o^ scaled to the reference conductance *G*_ref_. This maximum intercellular conductance is attained when the two cells are at the same polarized (*red*) or depolarized (*blue*) potentials. In addition to this *internal* voltage gating affecting the conductance *G_ij_*, the maximum conductance *G*^o^ could also be decreased by blocking the junction with *external* agents. The junction parameters are the potential *V*_0_ that determines the width of the bell-shaped function usually employed to fit the experimental distribution of conductances and the residual conductance and *G*_res_ (Spray et al., 1981; Cervera et al., 2018). The cases *V*_0_ = 18 mV and *G*_res_ = 0 **(A)** and *V*_0_ = 12 mV and *G*_res_ = 0.05*G*^o^ **(C)** are shown. The current-voltage curves of the junction [*right*, **(B,D)**] are obtained as the product *I* = *G_ij_*(*V_i_* − *V_j_*). These curves usually show a non-zero residual conductance [*right*, **(D)**] in the experimental range studied (Spray et al., 1981; Cervera et al., 2018).

TABLE S1 Distribution of various phenotypes in nicotine-exposed embryos.
