# Supplementary material for: HCN2 Channel-Induced Rescue of Brain Teratogenesis via Local and Long-Range Bioelectric Repair
Source: Front Cell Neurosci. 2020 May 26;14:136. doi: 10.3389/fncel.2020.00136 (PMC7264377; doi:10.3389/fncel.2020.00136)
Supplement: FIGURE S1 — The ion channel current (I)–voltage (V) curves show the outward and inward-rectifier currents and the total current (central curve) as a function of the voltage V for two effective channels (Gref = 1 nS; Cervera et al., 2014; Law and Levin, 2015; Cervera et al., 2018). (A) Two counteracting voltage gated channels and (B) a leakage channel counteracting a voltage-gated channel. The two stable (outer filled circles) and the unstable (central hollow circle) membrane potentials shown are particular values of the voltage V obtained from the condition of zero current, Vmem = V(I = 0). The stable values approximately correspond to the polarized and depolarized potentials assumed in these curves. In this model, the inward-rectifying channel of conductance Gpol acts to fix the polarized potential Epol while the outward-rectifying channel (A) or the leakage channel (B) of conductance Gdep favors the depolarized potential Edep, according to the approximate equation Vmem = (GpolEpol + GdepEdep)/(Gpol + Gdep); see Cervera et al. (2014); Law and Levin (2015); Cervera et al. (2018) and references therein for details. [file Data_Sheet_1.doc]

***Table 1: Distribution of various phenotypes in nicotine-exposed embryos***

| **Phenotypes in nicotine exposed embryos** | **Avg percentage across experiments** |
| --- | --- |
|  |  |
| Absent eyes, nostrils, forebrain, and majority of midbrain | 6.67% |
| Misformed eye, nostrils, forebrain, and midbrain | 47.58% |
| Misformed nostrils, forebrain, midbrain | 2.91% |
| Misformed brain with enlarged ventricles with brown fluid | 0.74% |
| Misformed eyes | 8.27% |
|  |  |
| All Phenotype | 66.20% |
| Normal tadpoles | 33.80% |
|  |  |
| Total | 100% |

***Supplementary Information* for the model of distant HCN2 channel expression mediating rescue of the nicotine-induced membrane voltage prepatterns disruption**

The case of a uniform expression HCN2-mediated rescue of nicotine-induced brain defects was studied in detail previously (Pai et al., 2018). Our main objective here is to suggest membrane voltage dynamics concerning the non-local, long-range effects of HCN2. In this approach, we give a qualitative view of the long-distance membrane voltage perturbation that is consistent with the experimental data concerning the position in relation to the neural plate region and the size of transplanted tissues.

To better understand the bioelectrical aspects of the problem, we used an electrically-focused approach (Kirkton and Bursac, 2011;Cervera et al., 2014;Law and Levin, 2015;Cervera et al., 2018) that allows a qualitative description of the long range effect. Voltage gated gap junctions have been shown to play important role in cell-cell communication during embryonic patterning (Spray et al., 1981;Warner, 1985;Pai et al., 2015;Mathews and Levin, 2016). The presence of functional gap-junctions in *Xenopus* embryos and their role in regulating cell communication has been documented and studied extensively (Warner, 1973;Warner et al., 1984;Warner, 1985;Levin and Mercola, 1998;2000;Cheng et al., 2002;Chernet et al., 2015). Gap-junctions are present throughout *Xenopus* embryonic development including neurulation (stage simulated here). Also, in our previous studies (Pai et al., 2015), we have shown functional role of GJs as an important component of how membrane voltage patterns regulate neural patterning. Hence, in this model we presumed cell-cell communication to occur via the intercellular gap-junctions. The model assumes a bell-shaped function for the voltage dependence of the gap junctions (GJs), in agreement with previous kinetic analysis of the voltage sensitivity of amphibian GJs where large transjunctional voltages give minimal conductance values (Harris et al., 1983) for the steady state conductance-voltage relation of the junctional membrane of amphibian blastomeres. Other models were used in (Pai et al., 2018). Based on the evidence as mentioned above we formulated functional GJs for the multicellular system described in the simulations, close to stage ~15 (neurulation) of Xenopus embryo. The results of Figure 5 concern only to the steady-state system states. Note also that a complete account of the GJs data and parameters used is given in the Figure of this file where different conductance-voltage curves for the GJ conductance *G_ij_*/*G*_ref_ between two neighboring cells *i* and *j* are obtained as a function of the potential difference *V_i_ – V_j_*.

As a first approximation to this complex channel interplay, the single-cell membrane voltage is determined by an inward-rectifying channel of voltage-gated conductance representing an *effective sum* of channels that promote the polarized (*pol*) cell state and an outward-rectifying channel of voltage-gated conductance representing the an *effective sum* of channels that promote the depolarized (*dep*) cell state (Kirkton and Bursac, 2011;Cervera et al., 2014;Law and Levin, 2015;Cervera et al., 2018). In these equations, *G*_pol_ and *G*_dep_ are the maximum channel conductances, *z* is the effective gating charge, *V*_th_ is the threshold potential, *V*_T_ is the thermal potential, and *V* is the cell potential (voltage). The channel currents can then be obtained as (pol channel), (dep channel), and $I_{leak}=G_{dep}V$ (leak channel), where *E*_pol_ and *E*_dep_ are the respective zero-current potentials (Hille, 1992). Because real-life channels may have mixed ionic conductances, the particular values for the potentials *E*_pol_ and *E*_dep_ are not easy to estimate. Examples representative of the general behavior obtained are given in Figure S1(a) for the channels used in the simulations and Figure S1(b) for the different case of a leakage channel. For completeness, Figure S2 shows the *pol* and *dep* voltage-gated conductances as a function of the voltage *V*.

**
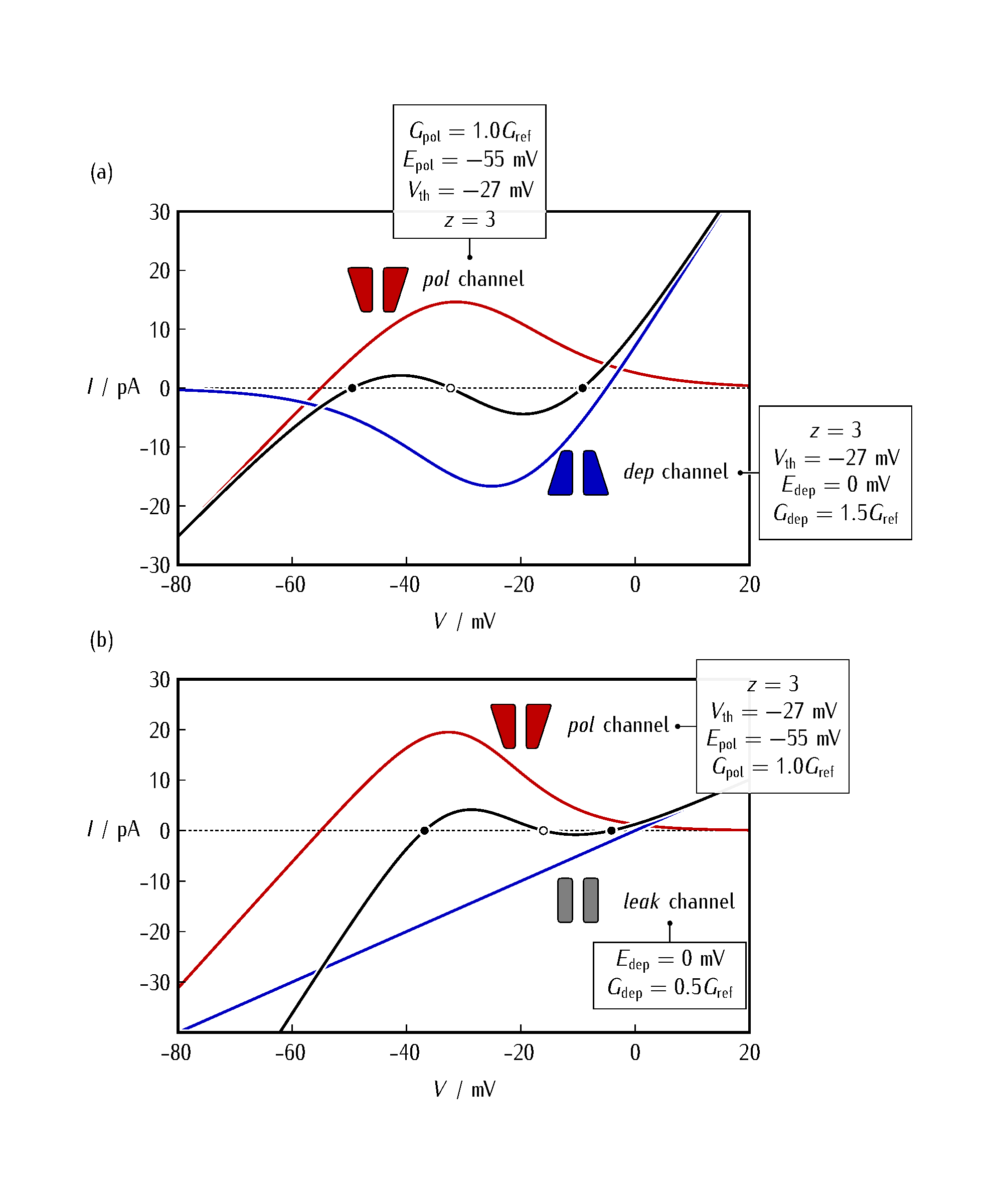
**

**Figure S1**. The ion channel current (*I*)–voltage (*V*) curves show the outward and inward-rectifier currents and the total current (central curve) as a function of the voltage *V* for two effective channels (*G*_ref_ = 1 nS) (Cervera et al., 2014;Law and Levin, 2015;Cervera et al., 2018). (a) two counteracting voltage gated channels and (b) a leakage channel counteracting a voltage-gated channel. The two stable (*outer filled circles*) and the unstable (*central hollow circle*) *membrane potentials* shown are particular values of the voltage *V* obtained from the condition of zero current, *V*_mem_ = *V*(*I* = 0). The stable values approximately correspond to the polarized and depolarized potentials assumed in these curves. In this model, the inward-rectifying channel of conductance *G*_pol_ acts to fix the polarized potential *E*_pol_ while the outward-rectifying channel (a) or the leakage channel (b) of conductance *G*_dep_ favors the depolarized potential *E*_dep_, according to the approximate equation *V*_mem_ = (*G*_pol_*E*_pol_ + G_dep_*E*_dep_)/(*G*_pol_ + *G*_dep_); see (Cervera et al., 2014;Law and Levin, 2015;Cervera et al., 2018) and references therein for details.

~~
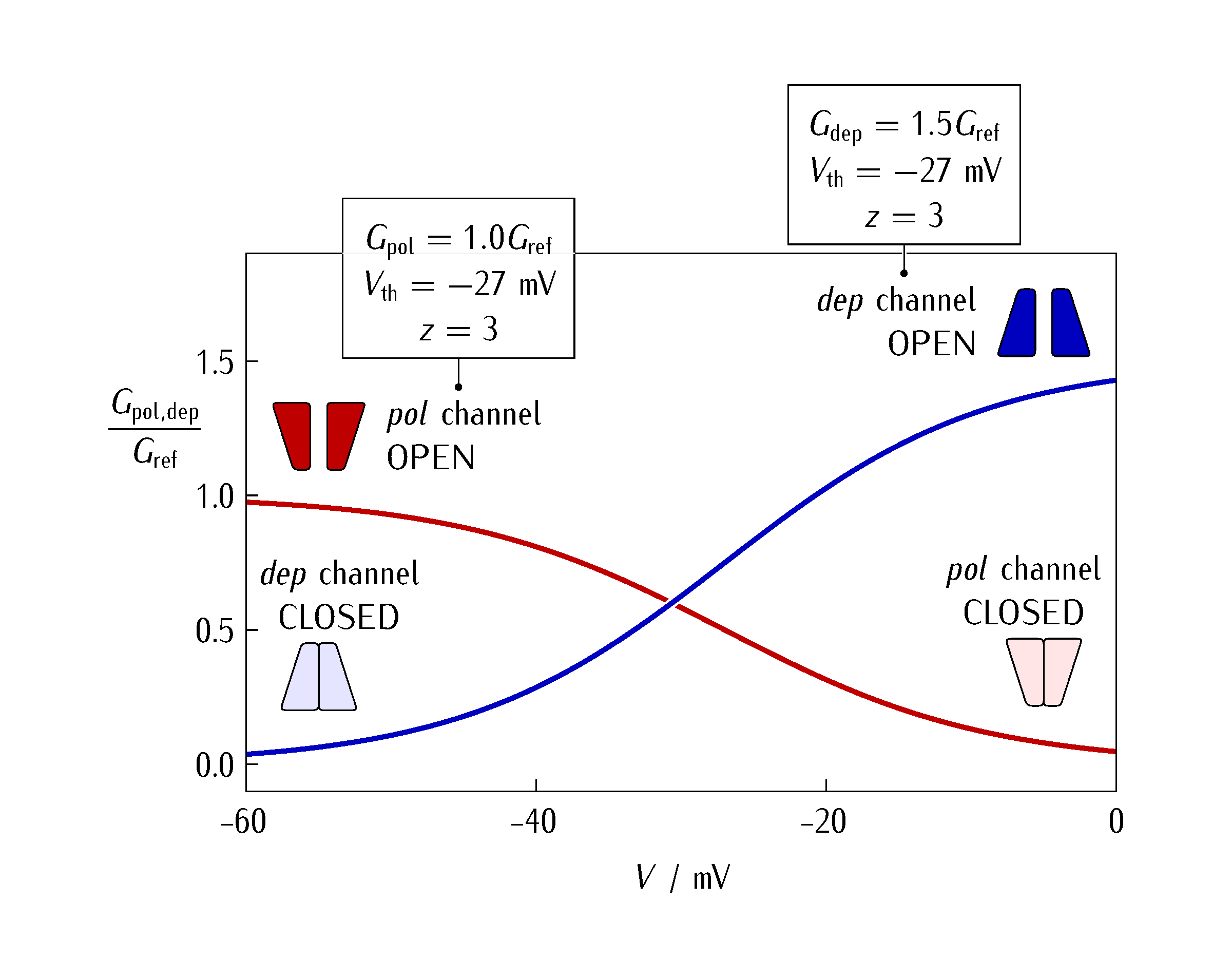
~~

**Figure S2**. The voltage-gated conductances as a function of the cell potential (voltage) *V*.

**Single Cell Simulation:**

Figure S1 shows the mathematical behavior for the cases of the two voltage-gated channels (a) and an alternative scheme that substitutes the *dep* voltage-gated channel for an omhic leakage channel (b). Note in particular that the concerted action of the HCN2 channels is simulated by an effective inward-rectifying channel whose conductance assumes non-zero values in the vicinity of the polarized potential *E*_pol_. While these channels conduct cations inwards at hyperpolarized potentials, they may show also a significant outwards conductance at more positive potentials, thus tending to stabilize the cell voltage near to the (resting) membrane potential. Thus, given the ionic gradients in *Xenopus* cells (high intracellular Na^+^ and K^+^ (Gillespie, 1983) in comparison to external media), the effective channel of maximum conductance *G*_pol_ may polarize rather than depolarize the cell (Pai et al., 2018), except for the case of strongly depolarized cells where this channel closes giving an almost zero residual conductance (Figures S1 and S2).

HCN channels interact with other voltage-dependent channels because of the strong coupling provided by the common cell membrane voltage. Note also that these HCN channels cannot establish the same voltage for every cell because different regions may show distinct ion channel (and thereby distinct membrane voltage) profiles. All these complexities suggest that simple approaches to the problem can be useful complements to more rigorous theoretical models (Pai et al., 2018), especially for a qualitative description of the membrane voltage dynamics in the system. The theoretical approach also assumes the existence of an effective ion channel of maximum conductance G_dep_ that promotes the depolarized cell state and counteracts the action of the channel that favors the polarized state. This *dep* channel is also a voltage-gated channel in the simulations presented [Figure S1(a)]*.*

Remarkably, Figure S1 suggests that a membrane potential bistability can exist at the single-cell level, i.e., without intercellular gap junctions, because of the opposed voltage behavior of the channels (Kirkton and Bursac, 2011;Cervera et al., 2014;Law and Levin, 2015;Cervera et al., 2018;Cervera et al., 2019). We also note that qualitatively similar results could be achieved with different choices for the single-cell complement of channels provided that the counterbalancing effects of the two generic channels acting to polarize and depolarize the cell are appropriately incorporated in the model (Kirkton and Bursac, 2011;Cervera et al., 2014;Law and Levin, 2015;Cervera et al., 2018;Cervera et al., 2019). This makes our model broadly applicable even beyond this study for understanding the dynamics of membrane voltage patterns in various tissues and conditions. It is important to note that: (*i*) the bistability of Figure S1(a) arises because of the voltage dependence of the *pol* and *dep* channels shown in Figure S2 (Kirkton and Bursac, 2011;Cervera et al., 2014;Law and Levin, 2015;Cervera et al., 2018;Cervera et al., 2019) and (*ii*) this bistability only occurs for a range of the maximum conductances ratio Gpol/Gdep. This question has been explained in more detail previously (Kirkton and Bursac, 2011;Cervera et al., 2014;Law and Levin, 2015;Cervera et al., 2018;Cervera et al., 2019).

**Multicellular Ensemble Simulation:**

The cells in multicellular ensemble are interconnected by gap junction channels that act as bioelectrical transistors with voltage-gated conductance (Spray et al., 1981;Cervera et al., 2018;Cervera et al., 2019). This intercellular coupling is weak enough to allow the multicellular regionalization of the electric potential, thus avoiding the limiting cases of no coupling (isolated cells) and strong coupling (equipotential multicellular ensemble where no patterning information can be stored) (Cervera et al., 2016a;Cervera et al., 2016b;2017). As to the mathematical behavior of the voltage-gated gap junctions, Figure S3 shows different examples of the intercellular conductance $G_{ij}=G_{\text{res}}+2G^{0}/\left\{1+\cosh[(V_{i}-V_{j})/V_{0}]\right\}$ as a function of the neighboring cell voltage difference (*V_i_ – V_j_*) where *V*_0_ characterizes the width potential of the curves.


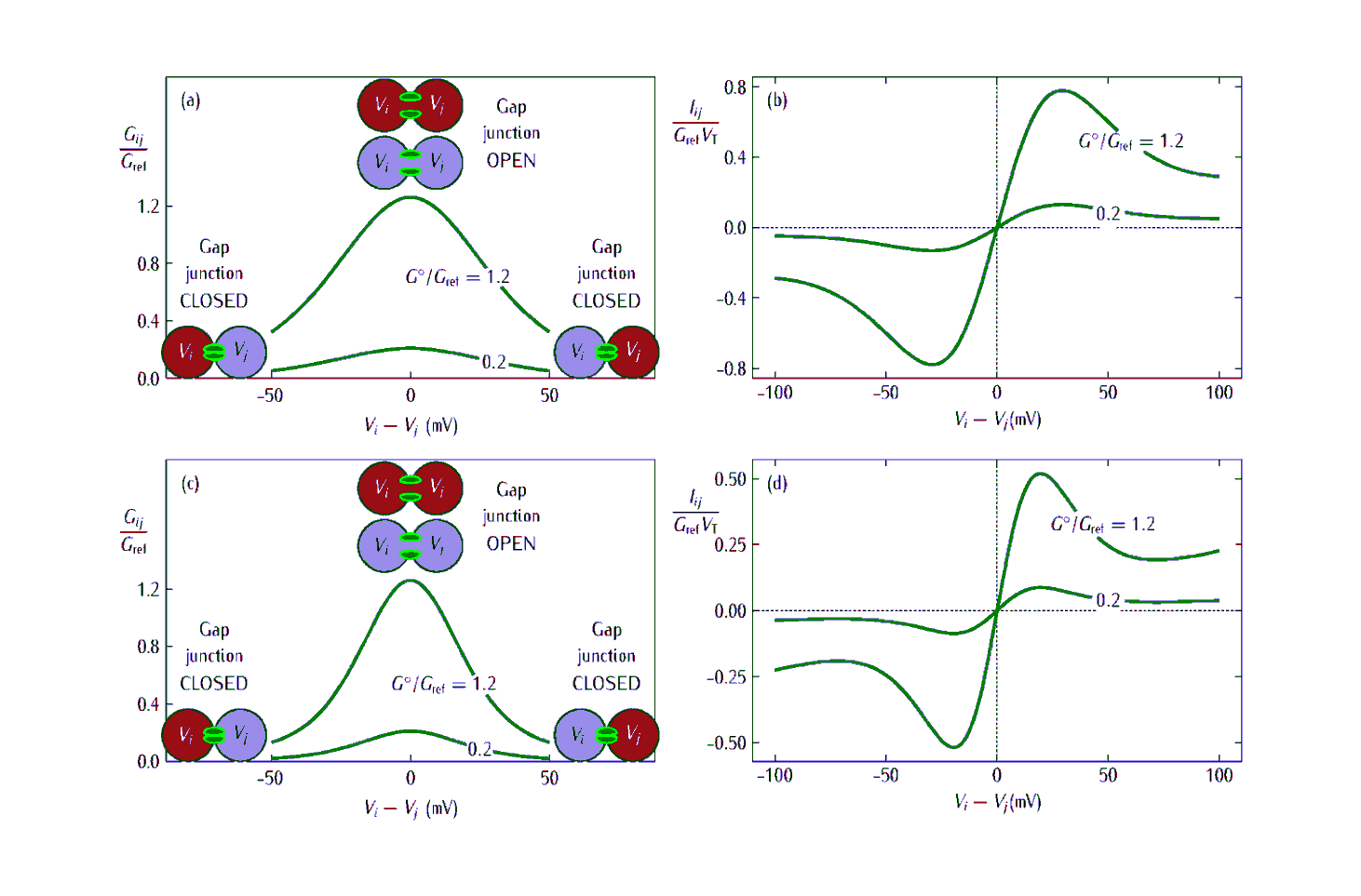


**Figure S3.** Experimentally, the gap junction conductance *G_ij_*/*G*_ref_ between two neighboring cells *i* and *j* follows a bell-shaped function of the difference *V_i_* – *V_j_* (*left*, (a) and (c)) which confers the intercellular plasticity (*right*, (b) and (d)) of the effective nodes in the multicellular ensemble. The curves show two high and low values of the maximum conductance *G*^o^ scaled to the reference conductance *G*_ref_. This maximum intercellular conductance is attained when the two cells are at the same polarized (*red*) or depolarized (*blue*) potentials. In addition to this *internal* voltage gating affecting the conductance *G_ij_*, the maximum conductance *G*^o^ could also be decreased by blocking the junction with *external* agents. The junction parameters are the potential *V*_0_ that determines the width of the bell-shaped function usually employed to fit the experimental distribution of conductances and the residual conductance and *G*_res_ (Spray et al., 1981;Cervera et al., 2018). The cases *V*_0_ = 18 mV and *G*_res_ = 0 (a) and *V*_0_ = 12 mV and *G*_res_ = 0.05*G*^o^ (c) are shown. The current-voltage curves of the junction (*right*, (b) and (d)) are obtained as the product *I* = *G_ij_*(*V_i_*- *V_j_*). These curves usually show a non-zero residual conductance (*right*, (d)) in the experimental range studied (Spray et al., 1981;Cervera et al., 2018).

Figures S1 and S3 show that the voltage *V*_i_ of cell i can change because of the single-cell channel currents *I*_pol,i_ and *I*_dep,i_ and the intercellular current regulated by *G*_ij_ and *V*_i_ – *V*_j_, which is calculated for the nearest neighboring cells around the central cell i (Cervera et al., 2016b;Cervera et al., 2018). The equations used for the currents *I*_pol,i_ and *I*_dep,i_ of Figure S1(a) qualitatively describe the observed experimental trends in terms of a small number of phenomenological parameters. In our model, we assume the effective charge *z* = 3 and the threshold potential *V*_th,pol_ = –*V*_T_ for the voltage-gated channels, with *V*_T_ = *RT*/*F* = 27 mV the thermal potential, where *R* is the gas constant, *T* is the temperature, and *F* is the Faraday constant. Also, the average number of neighboring cells is 4.

A key characteristic of the model is that the *single-cell state* can be modulated at the *ensemble level* because of the coupling of the central cell with the neighboring cells (Cervera et al., 2018). This coupling is allowed by the intercellular gap junction conductance *G*_ij_ that permits the transference of electric currents and signaling molecules (Cervera et al., 2017) between two adjacent cells. In particular, every cell experiences an *average electric potential* due to its nearest-neighbor cells. If the single-cell and intercellular coupling conductances are expressed in terms of a common reference value *G*_ref_, then large values of *G*^o^/*G*_ref_ give isopotential multicellular regions while low values of *G*^o^/*G*_ref_ give isolated cells with no bioelectrical communication.

The *N* cells in the multicellular ensemble are assumed to form an elliptic monolayer initially at the same voltage *V*_i_(*t* = 0), with i = 1, . . . , *N*. The evolution of the system for time *t* > 0 is given by the *N* equations for the cell voltages (Cervera et al., 2018). The ratio *C*_i_/*G*_pol_ gives an electrical time single-cell response lower than 1 s for capacitances and conductances in the ranges *C*_i_ = 10–100 pF and *G*_pol_ = 0.1–1 nS (Cervera et al., 2018), respectively. Note that this electrical time may increase significantly for the multicellular system if the intercellular conductance allowing the establishment of the spatio-temporal patterns takes low values. In contrast, the cell’s genetic processes not considered here are *relatively slow* because transcription and translation rate constants within the range 0.1–1 min^−1^ give times between 1 and 10 min while degradation rate constants within the range 0.003–0.1 min^−1^ give times between 0.1 and 5 h (Cervera et al., 2016b;Cervera et al., 2018). This is also the case of typical diffusional times *L*^2^/*D*, which are of the order of 1 h for a length *L* = 10^-3^ m (about 100 cells) and a diffusion coefficient *D* = 10^-10^ m^2^/s. Given the absence of genetic and biochemical components in our model we make no conclusions on involvement of genetic and biochemical signals directly or indirectly in this long-range action. Also, given that our model is not to scale in terms of number of cells and time of action we make no conclusions on the absolute distance or absolute time required for long-range action. The model focuses solely on changes in membrane voltage patterns. While the membrane voltage regionalizations obtained take times of tens of seconds because of the reduced number of cells considered, the final images included in the simulations correspond to 10^4^ s to check that the system has reached the steady-state. The input parameters used in each simulated condition can be found in the respective *Tables* below and have been justified previously.

**Simulation 1 – Controls (Figure 5A)**

| Bulk of the ensemble simulating *ventral/non-neural* region | | Patch simulating the *dorsal/neural* region | |
| --- | --- | --- | --- |
| *pol* conductance | *dep* conductance | *pol* conductance | *dep* conductance |
| *E*_pol_ = –55 mV | *E*_dep_ = –5 mV | *E*_pol_ = –55 mV | *E*_dep_ = –5 mV |
| *z* = 3 | *z* = 3 | *z* = 3 | *z* = 3 |
| *G*_pol_/*G*_ref_ = 1.0 | *G*_dep_/*G*_ref_ = 1.5 | *G*_pol_/*G*_ref_ = 1.8 | *G*_dep_/*G*_ref_ = 1.5 |
| *V*_th,pol_ = –27 mV | *V*_th,dep_ = –27 mV | *V*_th,pol_ = –27 mV | *V*_th,dep_ = –27 mV |
| 3 states are possible in this region:  *V_mem_ =* –49.5 mV (pol) , –32.3 mV (unstable), –9.2 mV (dep) | | only 1 state is possible in this region:  *V*_mem_ = –52.7 mV (pol) | |

**Cell capacitance:** *C* = 0.1 nF. **Maximum coupling intercellular conductances:** *G*^o^/*G*_ref_ =0.5. **Total number of cells in the ensemble:** 334 cells. **Initial (zero time) cell potentials:** *V*_mem, i_ (*t* = 0) = –9.2 mV for all cells. Total time for system evolution: 10^4^ s.

In this case, the bulk of the ensemble simulating the non-neural region has three possible the values of the membrane potential *V*_mem_ but it is assumed to be in the depolarized state. The neural region has only one possible polarized state. There is a clear interface (border – closed gap-junctions) between the polarized neural region and the depolarized non-neural region because of the bell-shaped function of *G*_ij_ (intercellular connectivity) that takes small values when the argument *V*_i_ – *V*_j_ of this function take high values (Cervera et al., 2018). As a consequence, the polarization of the neural region cannot proceed to the ventral region, being confined to the dorsal region only.

**Simulation 2 – Nicotine (Figure 5B)**

| Bulk of the ensemble simulating *ventral/non-neural* region | | Patch simulating the *dorsal/neural* region | |
| --- | --- | --- | --- |
| *pol* conductance | *dep* conductance | *pol* conductance | *dep* conductance |
| *E*_pol_ = –55 mV | *E*_dep_ = –5 mV | *E*_pol_ = –55 mV | *E*_dep_ = –5 mV |
| *z* = 3 | *z* = 3 | *z* = 3 | *z* = 3 |
| *G*_pol_/*G*_ref_ = 1.0 | *G*_dep_/*G*_ref_ = 1.5 | *G*_pol_/*G*_ref_ = 1.4 | *G*_dep_/*G*_ref_ = 1.5 |
| *V*_th,pol_ = –27 mV | *V*_th,dep_ = –27 mV | *V*_th,pol_ = –27 mV | *V*_th,dep_ = –27 mV |
| 3 states are possible in this region:  *V_mem_* = –49.5 mV (pol) , –32.3 mV (unstable), –9.2 mV (dep) | | 3 states are possible in this region:  *V_mem_ =* –51.8 mV (pol) , –19.9 mV (unstable), –14.2 mV (dep) | |

**Cell capacitance:** *C* = 0.1 nF. **Maximum coupling intercellular conductances:** *G*^o^/*G*_ref_ =0.5. **Total number of cells in the ensemble:** 334 cells. **Initial (zero time) cell potentials:** *V*_mem, i_ (*t* = 0) = –9.2 mV for all cells. Total time for system evolution: 10^4^ s.

The depolarizing action of nicotine is simulated by lowering the conductance *G*_pol_ in the neural region from *G*_pol_/*G*_ref_ = 1.8 to *G*_pol_/*G*_ref_ = 1.4 so that this region has now three possible values for *V*_mem_ instead of one (simulation 1), one of which is depolarized. This fact prevents the neural region from reaching the polarized state of simulation 1. Note that the depolarized non-neural region can also contribute to the stability of the now depolarized neural region. No additional nicotine effect on the gap junctions is assumed (*G*^o^/*G*_ref_ is kept fixed at 0.5).

**Simulation 3 – Nicotine + HCN2 (Figure 5C and D)**

| Bulk of the ensemble simulating *ventral/non-neural* region | | Patch simulating the *dorsal/neural* region | |
| --- | --- | --- | --- |
| *pol* conductance | *dep* conductance | *pol* conductance | *dep* conductance |
| *E*_pol_ = –55 mV | *E*_dep_ = –5 mV | *E*_pol_ = –55 mV | *E*_dep_ = –5 mV |
| *z* = 3 | *z* = 3 | *z* = 3 | *z* = 3 |
| *G*_pol_/*G*_ref_ = 1.0 | *G*_dep_/*G*_ref_ = 1.5 | *G*_pol_/*G*_ref_ = 1.4 | *G*_dep_/*G*_ref_ = 1.5 |
| *V*_th,pol_ = –27 mV | *V*_th,dep_ = –27 mV | *V*_th,pol_ = –27 mV | *V*_th,dep_ = –27 mV |
| 3 states are possible in this region:  *V_mem_ =* –49.5 mV (pol) , –32.3 mV (unstable), –9.2 mV (dep) | | 3 states are possible in this region:  *V_mem_* = –51.8 mV (pol) , –19.9 mV (unstable), –14.2 mV (dep) | |

*Small patch in the ventral/non-neural* region simulating externally forced polarization by HCN2 channels

| *pol* conductance | *dep* conductance |
| --- | --- |
| *E*_pol_ = –55 mV | *E*_dep_ = –5 mV |
| *z* = 3 | *z* = 3 |
| *G*_pol_/*G*_ref_ = 1.8 | *G*_dep_/*G*_ref_ = 1.5 |
| *V*_th,pol_ = –27 mV | *V*_th,dep_ = –27 mV |
| only 1 state is possible in this region: *V*_mem_ = –52.7 mV (pol) | |

**Cell capacitance:** *C* = 0.1 nF. **Maximum coupling intercellular conductances:** *G*^o^/*G*_ref_ = 0.5. **Total number of cells in the ensemble:** 334 cells. **Initial (zero time) cell potentials:** *V*_mem, i_ (*t* = 0) = –9.2 mV for all cells. Total time for system evolution: 10^4^ s.

An externally forced polarization of the small patch in the non-neural region that represents the HCN2 channel expressing tissue is simulated by an increase of the channel conductance *G*_pol_/*G*_ref_ = 1.8 in this small patch with respect to the value *G*_pol_/*G*_ref_ = 1.0 (simulation 2) which prevails in the rest of the non-neural region. Note that: *i*) the neural region is more prone to polarization than the ventral region because of the higher polarizing channel conductance and *ii*) the bulk of the ventral region is depolarized and dynamically buffers against the spread of polarization.

Remarkably, this external action can repolarize the dorsal region (Figure 5C). However, this repolarization cannot occur if the externally forced polarized patch in the non-neural region is too small to significantly influence the distant neural region (Figure 5D), suggesting that non-local actions should depend on the relative sizes of the HCN2 channel expressing multicellular patch and the target neural region.

**Simulation 4 – Nicotine + HCN2 (Figure 5E and F)**

| Bulk of the ensemble simulating *ventral/non-neural* region | | Patch simulating the *dorsal/neural* region | |
| --- | --- | --- | --- |
| *pol* conductance | *dep* conductance | *pol* conductance | *dep* conductance |
| *E*_pol_ = –55 mV | *E*_dep_ = –5 mV | *E*_pol_ = –55 mV | *E*_dep_ = –5 mV |
| *z* = 3 | *z* = 3 | *z* = 3 | *z* = 3 |
| *G*_pol_/*G*_ref_ = 1.0 | *G*_dep_/*G*_ref_ = 1.5 | *G*_pol_/*G*_ref_ = 1.4 | *G*_dep_/*G*_ref_ = 1.5 |
| *V*_th,pol_ = –27 mV | *V*_th,dep_ = –27 mV | *V*_th,pol_ = –27 mV | *V*_th,dep_ = –27 mV |
| 3 states are possible in this region:  *V_mem_ =* –49.5 mV (pol) , –32.3 mV (unstable), –9.2 mV (dep) | | 3 states are possible in this region:  *V_mem_* = –51.8 mV (pol) , –19.9 mV (unstable), –14.2 mV (dep) | |

Small patch in the *ventral/non-neural* region simulating externally forced polarization by HCN2 channels

| *pol* conductance | *dep* conductance |
| --- | --- |
| *E*_pol_ = –55 mV | *E*_dep_ = –5 mV |
| *z* = 3 | *z* = 3 |
| *G*_pol_/*G*_ref_ = 1.8 | *G*_dep_/*G*_ref_ = 1.5 |
| *V*_th,pol_ = –27 mV | *V*_th,dep_ = –27 mV |
| only 1 state is possible in this region: *V*_mem_ = –52.7 mV (pol) | |

**Cell capacitance:** *C* = 0.1 nF. **Maximum coupling intercellular conductances:** *G*^o^/*G*_ref_ =0.5. **Total number of cells in the ensemble:** 334 cells. **Initial (zero time) cell potentials:** *V*_mem, i_ (*t* = 0) = –9.2 mV for all cells. Total time for system evolution: 10^4^ s.

As in simulation 3, the externally forced polarization of a small patch in the non-neural region that represents the HCN2 channel expressing tissue is simulated by an increase of the channel conductance from *G*_pol_/*G*_ref_ = 1.0 (rest of the non-neural region) to *G*_pol_/*G*_ref_ = 1.8 in the small patch. This external action can repolarize the initially depolarized neural region only if the polarized patch in the ventral region is sufficiently close to the neural region (Figures 5E and F). The close proximity is required because the HCN2 patch in the non-neural region should act on the distant neural region through the now polarized non-neural cells located between these regions.

In summary, the results of simulations 3 and 4 qualitatively suggest that the concerted action of single-cell multistability (Figure S1) and intercellular coupling (Figure S3) can allow different system attractors in a bioelectrical phase space where external perturbations can trigger shifts between multicellular states. In particular, simulations 3 and 4 (Figures 5C–5F) suggest that context-dependent non-local actions can repolarize the distant dorsal/neural region, in qualitative agreement with experiments. However, the simulations also show that these externally forced actions can be effective only within a *limited range of experimental conditions* involving the relative sizes of the patch and the distant target region, and the spatial proximity between them. Indeed, the externally polarized non-neural patch can repolarize the neural region only by forcing the cells between them to repolarize (Figures 5C and 5E). The spread of this polarization front is not possible for too small (Figure 5D) or too distant (Figure 5F) polarizing patches.

Note also the importance of the stability of the bioelectrical single-cell polarized/depolarized states in the patch and the target region, which depends on the initially depolarized cell voltage value and the ratio of the effective *pol* and *dep* channel conductances in the different spatial regions (Cervera et al., 2014;2016a;Cervera et al., 2017). It is in this sense that the final outcome produced by the HCN2 channel expressing tissue should be context-dependent. In addition, the degree of intercellular coupling that allows the propagation of the polarizing signal from the ventral patch to the neural region should also be important in practical applications (Cervera et al., 2016b;Cervera et al., 2018).

The numerical algorithms employed in the simulations are described in References (Cervera et al., 2016a;Cervera et al., 2018); in particular, see the *Appendix* of the latter reference for the finite differences schemes used. Due to the relatively large multicellular ensembles and long calculation times needed, the numerical schemes are implemented in *Fortran 95* computer programs. These programs are compiled with *GNU Fortran 6.3* in *Debian GNU/Linux 9*. All simulations are performed on an *Intel® Core™ i7-4790K CPU* at 4.00 GHz. More details concerning the computational schemes and environment can be obtained directly from the authors upon reasonable request.

REFERENCES CITED

Cervera, J., Alcaraz, A., and Mafe, S. (2014). Membrane potential bistability in nonexcitable cells as described by inward and outward voltage-gated ion channels. *J Phys Chem B* 118**,** 12444-12450.

Cervera, J., Alcaraz, A., and Mafe, S. (2016a). Bioelectrical Signals and Ion Channels in the Modeling of Multicellular Patterns and Cancer Biophysics. *Sci Rep* 6**,** 20403.

Cervera, J., Meseguer, S., and Mafe, S. (2016b). The interplay between genetic and bioelectrical signaling permits a spatial regionalisation of membrane potentials in model multicellular ensembles. *Sci Rep* 6**,** 35201.

Cervera, J., Meseguer, S., and Mafe, S. (2017). MicroRNA Intercellular Transfer and Bioelectrical Regulation of Model Multicellular Ensembles by the Gap Junction Connectivity. *J Phys Chem B* 121**,** 7602-7613.

Cervera, J., Pai, V.P., Levin, M., and Mafe, S. (2019). From non-excitable single-cell to multicellular bioelectrical states supported by ion channels and gap junction proteins: Electrical potentials as distributed controllers. *Prog Biophys Mol Biol*.

Cervera, J., Pietak, A., Levin, M., and Mafe, S. (2018). Bioelectrical coupling in multicellular domains regulated by gap junctions: A conceptual approach. *Bioelectrochemistry* 123**,** 45-61.

Cheng, S.M., Chen, I., and Levin, M. (2002). KATP channel activity is required for hatching in Xenopus embryos. *Developmental Dynamics* 225**,** 588-591.

Chernet, B.T., Fields, C., and Levin, M. (2015). Long-range gap junctional signaling controls oncogene-mediated tumorigenesis in Xenopus laevis embryos. *Frontiers in physiology* 5**,** 519.

Gillespie, J.I. (1983). The distribution of small ions during the early development of Xenopus laevis and Ambystoma mexicanum embryos. *J Physiol* 344**,** 359-377.

Harris, A.L., Spray, D.C., and Bennett, M.V. (1983). Control of intercellular communication by voltage dependence of gap junctional conductance. *J Neurosci* 3**,** 79-100.

Kirkton, R.D., and Bursac, N. (2011). Engineering biosynthetic excitable tissues from unexcitable cells for electrophysiological and cell therapy studies. *Nat Commun* 2**,** 300.

Law, R., and Levin, M. (2015). Bioelectric memory: modeling resting potential bistability in amphibian embryos and mammalian cells. *Theor Biol Med Model* 12**,** 22.

Levin, M., and Mercola, M. (1998). Gap junctions are involved in the early generation of left-right asymmetry. *Dev Biol* 203**,** 90-105.

Levin, M., and Mercola, M. (2000). Expression of connexin 30 in Xenopus embryos and its involvement in hatching gland function. *Developmental Dynamics* 219**,** 96-101.

Mathews, J., and Levin, M. (2016). Gap junctional signaling in pattern regulation: Physiological network connectivity instructs growth and form. *Dev Neurobiol*.

Pai, V.P., Lemire, J.M., Pare, J.F., Lin, G., Chen, Y., and Levin, M. (2015). Endogenous Gradients of Resting Potential Instructively Pattern Embryonic Neural Tissue via Notch Signaling and Regulation of Proliferation. *J Neurosci* 35**,** 4366-4385.

Pai, V.P., Pietak, A., Willocq, V., Ye, B., Shi, N.Q., and Levin, M. (2018). HCN2 Rescues brain defects by enforcing endogenous voltage pre-patterns. *Nat Commun* 9**,** 998.

Spray, D.C., Harris, A.L., and Bennett, M.V. (1981). Equilibrium properties of a voltage-dependent junctional conductance. *J Gen Physiol* 77**,** 77-93.

Warner, A.E. (1973). The electrical properties of the ectoderm in the amphibian embryo during induction and early development of the nervous system. *J Physiol* 235**,** 267-286.

Warner, A.E. (1985). The role of gap junctions in amphibian development. *J Embryol Exp Morphol* 89 Suppl**,** 365-380.

Warner, A.E., Guthrie, S.C., and Gilula, N.B. (1984). Antibodies to gap-junctional protein selectively disrupt junctional communication in the early amphibian embryo. *Nature* 311**,** 127-131.
